# Construction and characterization of chimeric FcγR T cells for universal T cell therapy

**DOI:** 10.1186/s40164-025-00595-x

**Published:** 2025-01-15

**Authors:** Juanjuan Zhao, Manling Chen, Xudong Li, Zhaoqi Chen, Wei Li, Rongqun Guo, Min Wang, Zhongxing Jiang, Yongping Song, Jianxiang Wang, Delong Liu

**Affiliations:** 1 Department of Hematology, The First Affiliated Hospital of Zhengzhou University, Zhengzhou, Henan 450052 China; 2 State Key Laboratory of Experimental Hematology, Institute of Hematology and Blood Diseases Hospital, Chinese Academy of Medical Sciences & Peking Union Medical College, Tianjin, 300020 China; 3 Tianjin Key Laboratory of Cell Therapy for Blood Diseases, Institute of Hematology and Blood Diseases Hospital, Chinese Academy of Medical Sciences & Peking Union Medical College, Tianjin, China; 4 National Clinical Research Center for Blood Diseases, Institute of Hematology and Blood Diseases Hospital, Chinese Academy of Medical Sciences & Peking Union Medical College, Tianjin, China; 5 Department of Medicine, New York Medical College and Westchester Medical Center, Valhalla, NY USA

**Keywords:** FcγR, Universal CAR T, Off-the-shelf CAR T, Rituximab, Herceptin, Lymphoma, Ovarian cancer, Breast cancer

## Abstract

**Background:**

Several approaches are being explored for engineering off-the-shelf chimeric antigen receptor (CAR) T cells. In this study, we engineered chimeric Fcγ receptor (FcγR) T cells and tested their potential as a versatile platform for universal T cell therapy.

**Methods:**

Chimeric FcγR (CFR) constructs were generated using three distinct forms of FcγR, namely CD16A, CD32A, and CD64. The functionality of CFR T cells was evaluated through degranulation assays, specific target lysis experiments, in vitro cytokine production analysis, and assessment of tumor xenograft destruction specificity in mouse models using different monoclonal antibodies (MoAbs).

**Results:**

Three types of CFR T cells were engineered, 16s3, 32-8a, 64-8a CFR T cells. In the presence of rituximab (RTX), cytotoxicity of all three types of CFR T cells against CD20^+^ Raji-wt, K562-CD20^+^, and primary tumor cells was significantly higher than that of the mock T cells (*P* < 0.001). When herceptin was used, all three types of CFR T cells exhibited significant cytotoxicity against HER2^+^ cell lines of SK-BR-3, SK-OV-3, and HCC1954 (*P* < 0.001). The cytotoxicity of 64-8a CFR T cells was significantly inhibited by free human IgG at a physiological dose (*P* < 0.001), which was not observed in 16s3, 32-8a CFR T cells. Compared to 32-8a CFR T cells, 16s3 CFR T cells exhibited more prolonged cytotoxicity than 32-8a CFR T cells (*P* < 0.01). In in vivo assays using xenograft models, 16s3 CFR T cells significantly prolonged the survival of mice xenografted with Raji-wt cells in the presence of RTX (*P* < 0.001), and effectively reduced tumor burden in mice xenografted with SK-OV-3 cells in the presence of herceptin (*P* < 0.05). No significant non-specific cytotoxicity of CFR T cells was found in vivo.

**Conclusion:**

The anti-tumor effects of the CFR T cells in vitro and in xenograft mouse models are mediated by specific MoAbs such as RTX and herceptin. The CFR T cells therefore have the features of universal T cells with specificity directed by MoAbs. 16s3 CFR T cells are chosen for clinical trials.

**Supplementary Information:**

The online version contains supplementary material available at 10.1186/s40164-025-00595-x.

## Background

Several chimeric antigen receptor (CAR) T cell products have gained regulatory approval in the treatment of hematologic malignancies [1–5]. In addition to CAR T therapy, monoclonal antibody (MoAb) is another important method for cancer immunotherapy [6–11]. The interaction between the fragment crystallizable (Fc) region of IgG and the Fc-gamma receptor (FcγR) plays a pivotal role in initiating the anti-tumor effects of MoAbs [12–15].

Six FcγRs have been identified in humans, namely FcγRI, FcγRIIA, FcγRIIB, FcγRIIC, FcγRIIIA and FcγRIIIB. These receptors exhibit varying Fc binding affinities and trigger diverse functions such as antigen presentation, antibody-dependent cell-mediated cytotoxicity (ADCC), antibody-dependent cellular phagocytosis (ADCP), and immunosuppression [16–18]. The FcγRI (CD64) exhibits exclusive high affinity for monovalent Fc, whereas the low-affinity receptors, FcγRII (CD32) and FcγRIII (CD16), demonstrate effective binding solely to multivalent IgG Fc [19–21]. The CD32 family is encoded by three highly homologous genes, *CD32A*, *CD32B*, and *CD32C*, with their extracellular regions exhibiting a similarity exceeding 90% [22–24]. The CD32B receptor has been identified as the sole FcγR capable of transmitting inhibitory signals via immunoreceptor tyrosine inhibitory motifs [25]. The CD16B isoform represents the membrane-bound variant of CD16A [26].

Single nucleotide polymorphisms (SNPs) of Fc have been reported to affect the binding affinity to FcγRs and subsequently influence the therapeutic response of MoAbs [27, 28]. The available evidence suggests that the CD16A-158 V and CD32A-131 H variants, harboring SNPs with increased FcγR-Fc affinity, have the potential to augment the efficacy of rituximab, trastuzumab, and cetuximab [27–30].

Chimeric FcγR (CFR) T cells have been studied previously [31–37]. However, a consensus regarding the superior clinical translational potential of a specific type of CFR T cell has not yet been reached. In this study, we constructed CFRs incorporating the extracellular regions of FcγRs and engineered CFR T cells. The anti-tumor efficacy of CFR T cells against both hematological malignancies and solid tumors was evaluated both in vitro and in vivo.

## Materials and methods

### Cell lines

Raji-wt, K562-wt, HEK 293T, and MCF-7 cell lines were available in our laboratory. SK-OV-3 and SK-BR-3 cell lines were purchased from the American Type Culture Collection. The HCC1954 cell line was a gift from ImmuneOnco Biomedical Technology (Shanghai) Co., Ltd. The Raji-CD20^−/−^ cell line was generated by gene editing of Raji-wt cells using CRISPR/Cas9 [38]. K562-wt cells were transfected with a lentivirus vector containing a human CD20 sequence to generate K562-CD20^+^ cells [38]. The Raji-wt, K562-wt, Raji-CD20^−/−^, K562-CD20^+^, and HCC1954 cells were cultured in RPMI Medium-1640 basic (1×) (Gibco, USA) supplemented with 10% FBS (Gibco, USA). The SK-OV-3 cells were cultured in McCoy’s 5 A Medium (modified, 1×) (Gibco, USA) containing 10% FBS. The HEK 293T, SK-BR-3, and MCF-7 cells were cultured in Dulbecco’s Modified Eagle Medium (DMEM) basic (1×) (Gibco, USA) supplemented with 10% FBS. Adherent cells were detached using 0.25% trypsin solution (Gibco, USA) for 2–4 min prior to subsequent treatment.

### Patients and samples

Peripheral blood samples were collected from healthy adult subjects at the Tianjin Blood Center, following established laboratory protocols for T cell isolation, culture, and transduction [39–41]. B cells were extracted from the peripheral blood of healthy donors following the instruction using a B cell extraction kit (EasySep™ Human B Cell Isolation Kit, Cat# 17954, Stemcell Technologies). The peripheral blood and bone marrow specimens were collected from patients at the Institute of Hematology and Blood Diseases Hospital, Chinese Academy of Medical Sciences & Peking Union Medical College (IHBDH, CAMS&PUMC) in Tianjin, China. All subjects provided informed consent in accordance with the ethical guidelines (Declaration of Helsinki) and received approval from the ethical advisory board of IHBDH, CAMS&PUMC. Primary tumor cells were cultured in Iscove’s modified Dulbecco’s medium (IMDM, Gibco, USA) supplemented with 15% FBS, 100 ng/ml rhFLT3-L (Cat. #300 − 19, PeproTech, USA), 100 ng/ml rhSCF (Cat. #300-07, PeproTech, USA) and 50 ng/ml rhTPO (Cat. #300 − 18, PeproTech, USA).

### Plasmid construction, lentivirus production, and CFR T cell preparation

The cDNA sequences of the human CD16A-158 V, CD32A-131 H, and CD64 extracellular domains were retrieved from the gene bank (https://www.ncbi.nlm.nih.gov/gene/) and the Uniprot database (https://www.uniprot.org). The cDNAs were synthesized by Tsingke Biotechnology Co., Ltd., Beijing. The aforementioned sequences were incorporated into a previously constructed pCDH-CAR plasmid harboring a CD8α hinge and transmembrane domain, as well as an intracellular domain of 4-1BB-CD3ζ-T2A-GFP [39–41]. Lentivirus packaging was conducted as previously described [38]. The backbone plasmid and packaging plasmids (pRSV-Rev, pMDLg/pRRE, and pMD2.G) were added into the culture dish of HEK293 T cells at a molar ratio of 3:1:1:1. After 48 h, the supernatant was collected and centrifuged for concentration at 50,000 g for 2 h (4 °C). The isolated human primary T cells were transduced with virus particles 24 h after activation using Dynabeads™ Human T-Activator CD3/CD28 (Cat. #11132D, Gibco, USA), followed by centrifugation at 1500 rpm for 1.5 h (32 °C) to obtain CFR T cells.

### Flow cytometry assays

The antibodies employed in flow cytometry assays for CAR expression, activation, subsets, exhaustion, and apoptosis of T cells are listed as follows:

**Table:** 

Antibody	Cat.	Clone	Manufacturer
APC anti-human CD16	302,012	3G8	Biolegend (USA)
APC anti-human CD32	303,208	FUN-2	Biolegend (USA)
APC anti-human CD64	305,014	10.1	Biolegend (USA)
Alexa Fluor^®^ 647 Annexin V	640,912	——	Biolegend (USA)
Propidium iodide (PI) solution	421,301	——	Biolegend (USA)
APC anti-human CD25	302,610	BC69	Biolegend (USA)
PE anti-human CD69	310,906	FN50	Biolegend (USA)
PerCP/Cyanine5.5 anti-human CD8	344,710	SK1	Biolegend (USA)
APC/Cyanine7 anti-human CD45RA	304,128	HI100	Biolegend (USA)
PE anti-human CD197 (CCR7)	353,204	G043H7	Biolegend (USA)
APC/Cyanine7 anti-human CD279 (PD-1)	329,922	EH12.2H7	Biolegend (USA)
PE anti-human TIGIT (VSTM3)	372,704	A15153G	Biolegend (USA)
PE anti-human CD366 (Tim-3)	345,006	F38-2E2	Biolegend (USA)
PE/Cyanine7 anti-human CD223 (LAG-3)	369,310	11C3C65	Biolegend (USA)

### MoAbs and immunoglobulin

RTX and herceptin were purchased from Roche. Sintilimab (PD-1 MoAb) was obtained from Xinda Bio Pharmaceutical (Hangzhou) Co., Ltd. Additionally, human Immunoglobulin (pH4) for intravenous injection was purchased from Yuanda Shuyang Pharmaceutical Co., Ltd.

### Cell proliferation assays

On the 3rd day post-transduction, T cell counting was conducted using a cytometer (CellDrop, DeNovix^®^). A total of 5 × 10^5^ T cells were transferred to a fresh 6-well plate. Counting was repeated every 3 days. To investigate the impact of the MoAb on proliferation, target cells were labelled with CFSE (CellTrace™ CFSE Cell Proliferation Kit, Cat. #C34554, Invitrogen™) following the manufacturer’s instructions.

### Degranulation assay

The CD107a serves as a sensitive marker for cell degranulation. A total of 5 × 10^4^ target cells were co-cultured with T cells at an effector-to-target (E: T) ratio of 1:1 in a volume of 200 µl added with PE anti-human CD107a (Cat. #328608, H4A3, Biolegend, USA). MoAbs rituximab (RTX) targeting CD20 and herceptin against HER2 were added to the respective cultures of the target cells at a concentration of 1 µg/ml. Monensin (Cat. #420701, Biolegend, USA) was introduced into the culture system at a final concentration of 2 µM after one hour. Subsequently, flow cytometry analysis was performed 4 hours later to determine the percentages of CD107a-positive CFR T cells.

### Target cell lysis by CFR T cells in vitro

Target cells at 1 × 10^5^/well were inoculated in a 24-well plate with either the CFR T cells or the mock T cells, supplemented with or without a specific MoAb. Adherent target cells were incubated for at least 4 h prior to seeding effector cells. Cells were harvested 24–48 h post-incubation. T cells were labelled with APC/Cyanine7-conjugated anti-human CD3 (Cat. #317342, OKT3, Biolegend), while target cells were labelled with PE-conjugated anti human CD20 (Cat. #302306, 2H7, Biolegend), PE-conjugated anti human CD19 (Cat. #392506, 4G7, Biolegend) or PE-conjugated anti human CD340 (erbB2/HER2) (Cat. #324406, 24D2, Biolegend) antibody, respectively. Flow cytometry was used to determine the lysis of target cells.

The cytotoxicity of CFR T cells on breast and ovarian cancer cell lines (SK-BR-3 and SK-OV-3) was confirmed using the real-time cellular analysis (RTCA). The RTCA (Agilent xCELLigence RTCA DP) is a real-time, impedance-based cytotoxicity assessment that can reflect the lysis of target cells through the cell index (CI) [42, 43]. The target cells were co-cultured with CFR T cells in a 96-well plate (Agilent E-plate 96), and the changes in CI values were monitored using the RTCA system.

### Cytokine level detection by ELISA

After 24 h of co-culturing the target cells with the effector cells, the supernatant was collected. ELISA kits from R&D Systems for human IFN-γ (Cat. #SIF50C), IL-2 (Cat. #S2050), TNF-α (Cat. #STA00D), and IL-6 (Cat. #S6050) were employed to detect the levels of cytokines.

### Repetitive antigenic stimulation experiment

To determine the long-term cytotoxic potential, CFR T cells were repetitively stimulated by the target antigen [44–46]. In the initial round (R1) of the repetitive stimulation, 1 × 10^5^ Raji-wt cells were co-cultured with effector T cells at an E: T ratio of 2:1 (R1-0). After an incubation period of 72 h (R1-72 h), cells were harvested for counting and flow cytometry analysis. Subsequently, re-plating was performed at an E: T ratio of 2:1 using effector cells from the preceding round.

### In vivo assay for cytotoxicities of CFR T cells

The mice (NOD/SCID mice and BALB/c nude mice) were purchased from the Animal Science Laboratory (CAMS&PUMC, China) and housed in a specific pathogen-free environment. All animal experiments were conducted with the approval of the Institutional Animal Care and Use Committees of Peking Union Medical College.

### Lymphoma transplantation model

On day 0, 40 female NOD/SCID mice aged 7 weeks were irradiated with a dose of 0.825 Gy and randomly allocated to 6 groups according to different treatment, ensuring consistent weight distribution within each group. Subsequently, 1 × 10^6^ tumor cells were injected through each tail vein 8 h later. Except for group Raji-CD20^−/−^ (*n* = 7), all other groups (group 16s3 CFR T-RTX, *n* = 7; mock T-RTX, *n* = 7; PBS-RTX, *n* = 7; 16s3 CFR T-herceptin, *n* = 6; mock T-herceptin, *n* = 6) were transplanted with Raji-wt cells. On days 4, 8 and 12, a total of 1 × 10^7^ effector cells or PBS combined with RTX or herceptin (60 µg) were administered via each tail vein. The mice’s body weight was monitored twice a week, and their overall survival period was recorded. Pathological analysis was conducted on the liver, spleen, and bone marrow of deceased mice.

### Ovarian cancer xenograft model

Twenty female BALB/c nude mice, aged 5–6 weeks, were exposed to a radiation dose of 2 Gy. Subsequently, they were evenly assigned into 4 groups (*n* = 5) randomly based on different treatment, ensuring consistent weight distribution within each group. Following an interval of 8 h, SK-OV-3 cells (5 × 10^6^/mouse) were subcutaneously inoculated in the right posterior back region. On days 8 and 14, 1 × 10^7^ effector cells combined with or without herceptin (30 µg) were administered via each tail vein. The weight of the mice and the size of tumors were measured twice a week. Tumor volumes were calculated using the formula *V* (mm^3^) = (*l* × *w*^2^) / 2, where *w* represents the width of the smaller tumor perpendicular axis, and *l* represents the length of the larger axis. On day 25, euthanasia was performed on all mice, followed by removing and weighing of tumor masses for pathological examination.

### Statistical analyses

A repeated measures ANOVA was used to compare the T cell proliferation and tumor volume between different groups. The differences among the specific killing, CD107a expression, CI value, cytokine level and tumor weight were analyzed using two-way ANOVA. A paired *t*-test was used to evaluate the effect of PD-1 MoAb on CFR T cells. The Kaplan-Meier test was used to estimate overall survival, and the log-rank test was used to compare differences in survival. The significance level of *P* < 0.05 indicates a statistically significant difference, * denotes *P* < 0.05, * * denotes *P* < 0.01, and *** denotes *P* < 0.001, while n.s. signifies no statistical difference.

## Results

### The structures of CFRs and characteristics of the CFR T cells

**Fig. 1:**
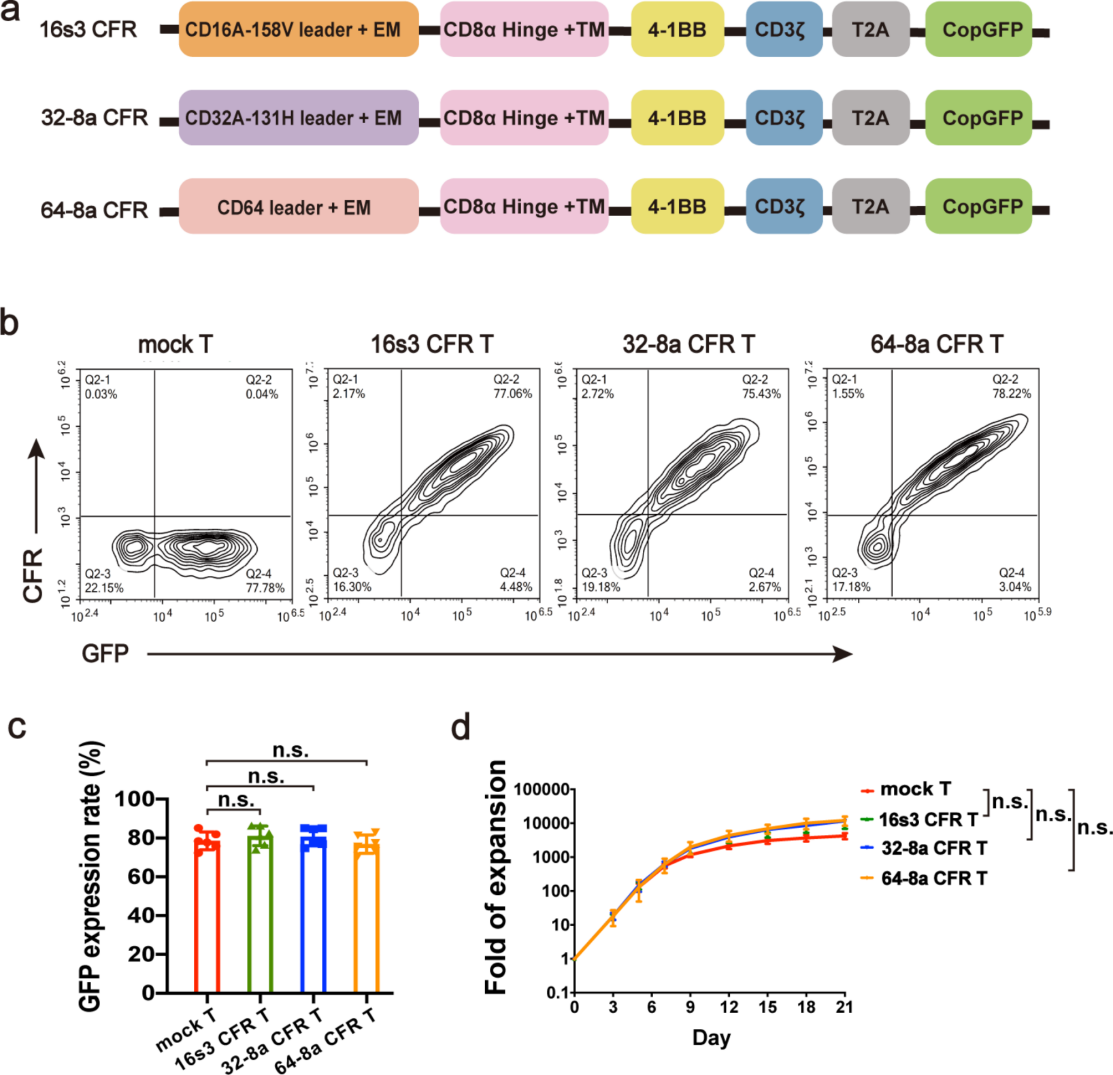
The structures of chimeric Fc-gamma receptors (CFRs), the expression of CFR on T cells and the proliferation of the CFR T cells. **a** The structures of 16s3, 32-8a and 64-8a CFR. **b** The flow cytometry diagram showing the infection efficiency of CFR T cells and the mock T cells. **c** The bar graph comparing the GFP expression percentages of the CFR T cells with that of the mock T cells at Day 5 post-infection determined by flow cytometry (*n* = 6; n.s., no significance). **d** The proliferation of 16s3, 32-8a, 64-8a CFR T cells and the mock T cells on Day 3, 5, 7, 9, 12, 15, 18, 21 (*n* = 6; n.s., no significance)

Three distinct CFRs were generated using different extracellular regions of human FcγR subtypes, namely 16s3, 32-8a, and 64-8a, respectively (Fig. 1a). The expression levels of CFRs and GFP of the transduced T cells (CFR T cells) were determined by flow cytometry on Day 5 post-infection (Fig. 1b, c). The expression percentages of CD16, CD32, CD64 on the 16s3, 32-8a, 64-8a CFR T cells were 73.98–86.33%, 74.81–85.01%, 70.66–82.99%, respectively. The expression percentages of CD16, CD32, CD64 on the T cells transduced with a mock construct lacking extracellular sequences (mock T cells) were 0.012–0.073%, 0–0.036%, 0–0.063%, respectively. The CFR T cells were further characterized for their proliferation potential. The cell proliferation after transduction was monitored by cell number at different days (Day 3, 5, 7, 9, 12, 15, 18, 21). No significant differences in proliferation were observed among various CFR T cells and the mock T cells (Fig. 1d). The exhaustion (PD-1, LAG3, TIGIT, TIM3), activation (CD25, CD69), and subpulations [CD8, CD4, naïve T (Tn), central memory T (Tcm), effector memory T (Tem), terminal effector T (Te)] during culture were also compared. Overall, no significant differences were observed despite variations between different samples (Fig. S1–3).

### The CFR T cells exhibited RTX-mediated specific cytotoxicity against human CD20+ cell lines and primary cells

The CD20-expressing cell lines (Raji-wt and K562-CD20^+^) and primary mononuclear cells were used as target cells (Fig. S4a, b). The primary mononuclear cells were collected from peripheral blood (PBMCs) or bone marrow (BMMCs) from 3 patients diagnosed respectively with mantle cell lymphoma (MCL), diffuse large B cell lymphoma (DLBCL) or chronic lymphocytic leukemia (CLL).

Both 16s3 and 32-8a CFR T cells exhibited superior cytotoxicity at RTX concentrations ≥ 1 µg/ml, whereas a RTX concentration of ≥ 0.1 µg/ml was optimal for 64-8a CFR T cells (Fig. S4c). Therefore, the 1 µg/ml concentration of RTX was chosen for subsequent investigations.

**Fig. 2:**
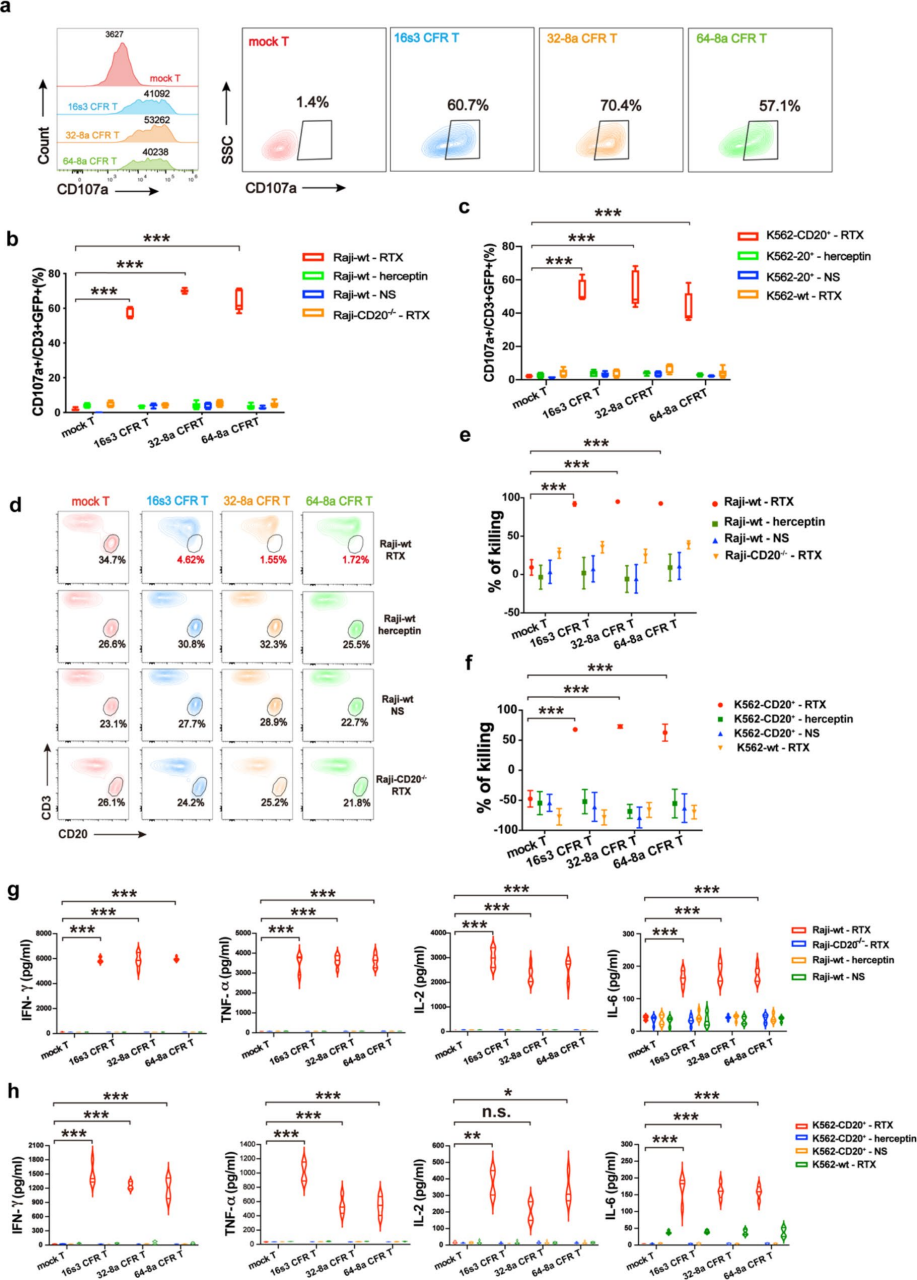
The rituximab (RTX)-mediated specific cytotoxicity of CFR T cells against CD20^+^ cell lines. **a** The flow cytometry diagram showing the elevated median fluorescence intensity (MFI) and percentages of CD107a in 16s3, 32-8a, 64-8a CFR T and mock T cells when co-cultured with Raji-wt cells in the presence of RTX (1 µg/ml). **b** The percentages of CD107a^+^ cells in 16s3, 32-8a, 64-8a CFR T and mock T cells when co-cultured with Raji-wt and Raji-CD20^−/−^ in the presence of RTX (1 µg/ml), herceptin (1 µg/ml) or normal saline (NS) (*n* = 6; ***, *P* < 0.001). **c** The percentages of CD107a^+^ cells in 16s3, 32-8a, 64-8a CFR T and mock T cells co-cultured with K562-CD20^+^ and K562-wt in the presence of RTX (1 µg/ml), herceptin (1 µg/ml) or NS (*n* = 6; ***, *P* < 0.001). **d** The flow cytometry diagram showing the residual Raji-wt and Raji-CD20^−/−^ cells after co-culturing with 16s3, 32-8a, 64-8a CFR T and mock T cells in the presence of RTX (1 µg/ml), herceptin (1 µg/ml) or NS for 24 h. **e** The cytotoxicity against Raji-wt and Raji-CD20^−/−^ by 16s3, 32-8a, 64-8a CFR T and mock T cells in the presence of RTX (1 µg/ml), herceptin (1 µg/ml) or NS after 24 h of co-culturing (E: T = 2:1; *n* = 4; ***, *P* < 0.001). **f** The cytotoxicity against K562-CD20^+^ and K562-wt by 16s3, 32-8a, 64-8a CFR T and mock T cells in the presence of RTX (1 µg/ml), herceptin (1 µg/ml) or NS after 24 h of co-culturing (E: T = 2:1; *n* = 4; ***, *P* < 0.001). **g** The cytokines released by 16s3, 32-8a, 64-8a CFR T and mock T cells co-cultured with Raji-wt and Raji-CD20^−/−^ in the presence of RTX (1 µg/ml), herceptin (1 µg/ml), or NS at 24 h (*n* = 6; ***, *P* < 0.001). **h** The cytokines released by 16s3, 32-8a, 64-8a CFR T and mock T cells co-cultured with K562-CD20^+^ and K562-wt in the presence of RTX (1 µg/ml), herceptin (1 µg/ml) or NS at 24 h (*n* = 4; ***, *P* < 0.001; **, *P* < 0.01; *, *P* < 0.05; n.s., no significance)

**Fig. 3:**
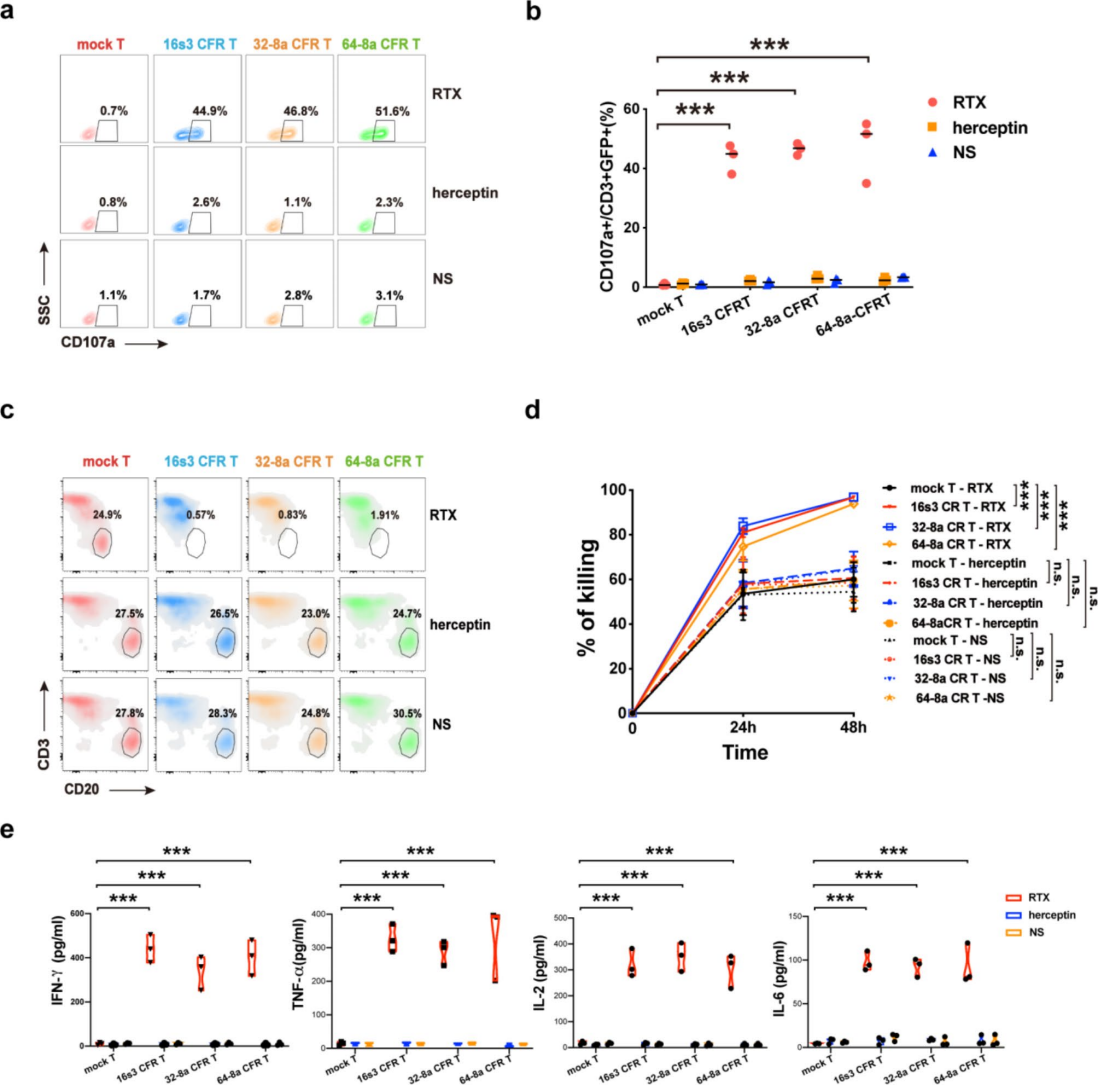
Rituximab (RTX)-mediated anti-tumor effect of CFR T cells on CD20^+^ primary tumor cells. **a** The flow cytometry diagram showing the levels of CD107a in 16s3, 32-8a, 64-8a CFR T and mock T cells when co-cultured with the CD20^+^ primary tumor cells in the presence of RTX (1 µg/ml), herceptin (1 µg/ml) or NS. **b** The percentages of CD107a^+^ cells in 16s3, 32-8a, 64-8a CFR T and mock T cells co-cultured with primary tumor cells in the presence of RTX (1 µg/ml), herceptin (1 µg/ml) or NS (*n* = 3; * * *, *P* < 0.001). **c** The flow cytometry diagram showing the residual CD20^+^ primary tumor cells after co-culturing with 16s3, 32-8a, 64-8a CFR T and mock T cells in the presence of RTX (1 µg/ml), herceptin (1 µg/ml) or NS for 48 h. **d** The cytotoxicity against CD20^+^ primary tumor cells by 16s3, 32-8a, 64-8a CFR T and mock T cells in the presence of RTX (1 µg/ml), herceptin (1 µg/ml) or NS (E: T = 2:1; *n* = 3; * * *, *P* < 0.001; n.s., no significance). **e** The cytokines released by 16s3, 32-8a, 64-8a CFR T and mock T cells co-cultured with CD20^+^ primary tumor cells in the presence of RTX (1 µg/ml), herceptin (1 µg/ml) or NS at 24 h (*n* = 3; ***, *P* < 0.001)

CD107a, also known as lysosomal-associated membrane protein 1 (LAMP-1), is a marker for T cell degranulation/activation. The CD107a level assay was employed to quantify degranulation and characterize the production of cytotoxic granules from the CFR T cells. Upon co-culturing the CFR T cells with Raji-wt, K562-CD20^+^ or CD20^+^ primary cells in the presence of RTX, a significant increase in the percentages of CD107a expression was observed in the CFR T cells when compared to those in the mock T cells (Figs. 2a–c and 3a and b). Similarly, the specific lysis of Raji-wt, K562-CD20^+^ or CD20^+^ primary cells by the CFR T cells in the presence of RTX was significantly increased (Figs. 2d–f and 3c and d). Furthermore, the cytokine levels of IFN-γ, TNF-α, IL-2 and IL-6 secreted by the CFR T cells increased dramatically when the CFR T cells and CD20^+^ target cells were co-cultured in the presence of RTX (Figs. 2g and h and 3e), with the exception for IL-2 secreted by 32-8a CFR T cells co-cultured with K562-CD20^+^ cells (Fig. 2h IL-2 panel). For the control groups treated with a non-specific MoAb (herceptin groups) or normal saline (NS), as well as co-cultured with the tumor cells without CD20 expression (Raji-CD20^−/−^ groups and K562-wt groups), no significant cytotoxicity of CFR T cells was observed (Figs. 2b–h and 3a–e). In addition to its action against CD20^+^ primary tumor cells, RTX also mediated the elimination of CD20^+^ healthy B cells by the CFR T cells (Fig. S5). These results indicate that the cytotoxicity of the CFR T cells against CD20^+^ tumor cell lines and primary cells were mediated by target-specific RTX.

### The CFR T cells showed herceptin-mediated specific cytotoxicity against HER2+ solid tumor cell lines

**Fig. 4:**
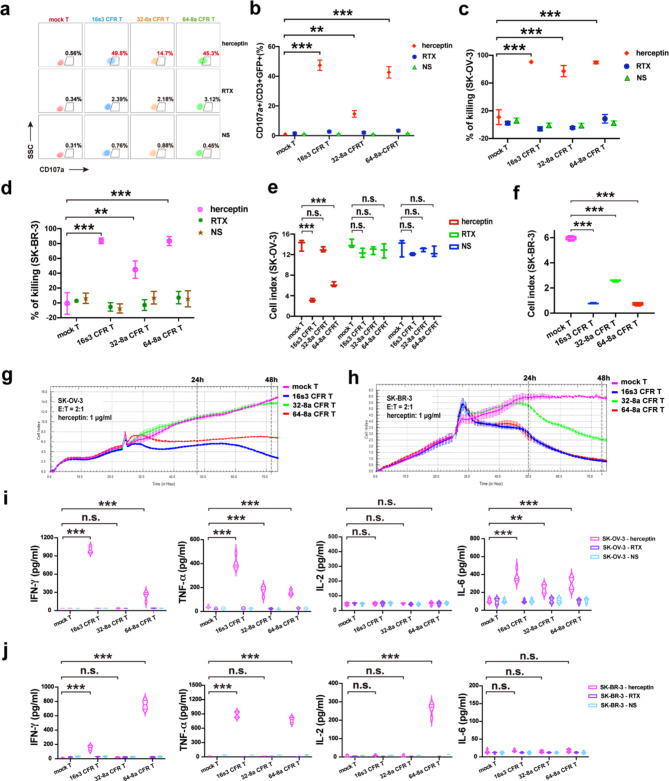
The specific cytotoxicity of CFR T cells towards HER2^+^ cell lines mediated by herceptin. **a** The flow cytometry diagram showing the percentages of CD107a expression on 16s3, 32-8a, 64-8a CFR T and mock T cells co-cultured with SK-OV-3 cells in the presence of herceptin (1 µg/ml), rituximab (RTX) (1 µg/ml) or NS. **b** The significant increase of CD107a expression on 16s3, 32-8a, 64-8a CFR T and mock T cells co-cultured with SK-OV-3 in the presence of herceptin (1 µg/ml), RTX (1 µg/ml) or NS (*n* = 5; * * *, *P* < 0.001; * *, *P* < 0.01). **c** The SK-OV-3 lysis by 16s3, 32-8a, 64-8a CFR T and mock T cells in the presence of herceptin (1 µg/ml), RTX (1 µg/ml) or NS at 48 h determined by flow cytometry (E: T = 2:1; *n* = 5; * * *, *P* < 0.001). **d** The SK-BR-3 lysis by 16s3, 32-8a, 64-8a CFR T and mock T cells in the presence of herceptin (1 µg/ml), RTX (1 µg/ml) or NS at 48 h determined by flow cytometry (E: T = 2:1; *n* = 5; * * *, *P* < 0.001; * *, *P* < 0.01). **e** The CI values of SK-OV-3 co-cultured with 16s3, 32-8a, 64-8a CFR T and mock T cells at the E: T ratio of 2:1 in the presence of herceptin (1 µg/ml), RTX (1 µg/ml) or NS at 48 h (*n* = 3; * * *, *P* < 0.001; n.s., no significance). **f** The CI values of SK-BR-3 co-cultured with 16s3, 32-8a, 64-8a CFR T and mock T cells at the E: T ratio of 2:1 in the presence of herceptin (1 µg/ml) at 48 h (*n* = 4; * * *, *P* < 0.001). **g** The CI values displayed by the RTCA system of SK-OV-3 co-cultured with 16s3, 32-8a, 64-8a CFR T and mock T cells at the E: T of 2:1 in the presence of herceptin (1 µg/ml). **h** The CI values displayed by the RTCA system of SK-BR-3 co-cultured with 16s3, 32-8a, 64-8a CFR T and mock T cells at the E: T of 2:1 in the presence of herceptin (1 µg/ml). **i** The cytokines released by 16s3, 32-8a, 64-8a CFR T and mock T cells co-cultured with SK-OV-3 and in the presence of herceptin (1 µg/ml), RTX (1 µg/ml) or NS at 24 h (*n* = 4; * * *, *P* < 0.001; * *, *P* < 0.01; n.s., no significance). **j** The cytokines released by 16s3, 32-8a, 64-8a CFR T and mock T cells co-cultured with SK-BR-3 in the presence of herceptin (1 µg/ml), RTX (1 µg/ml) or NS at 24 h (*n* = 4; * * *, *P* < 0.001; n.s., no significance)

HER2 expressing solid tumor cell lines (SK-OV-3 and SK-BR-3) were used as target cells (Fig. S6a). When co-cultured with SK-OV-3 cells, the CFR T cells showed significantly increased CD107a production in the presence of herceptin (Fig. 4a, b). The specific lysis of SK-OV-3 and SK-BR-3 by the CFR T cells in the presence of herceptin was substantially elevated in comparison to that by the mock T cells (Fig. 4c, d).

The RTCA system was utilized to estimate the specific lysis of adherent target cells, as indicated by CI values [42, 43]. A lower CI value indicates higher cytotoxicity. Remarkably reduced CI values were observed for SK-OV-3 co-cultured with 16s3 and 64-8a CFR T cells (E: T = 2:1) in the presence of herceptin (Fig. 4e, g). However, no significant reduction in CI values was observed for SK-OV-3 co-cultured with 32-8a CFR T cells mediated by herceptin (Fig. 4e, g).

To confirm this target-specific lysis, we examined another cell line SK-BR-3. Similarly, significant reductions in CI values of SK-BR-3 were found when co-cultured with 16s3 and 64-8a CFR T cells in the presence of herceptin (Fig. 4f, h). Additionally, the CI values of SK-BR-3 cells also decreased significantly when co-cultured with 32-8a CFR T cells in the presence of herceptin at 48 h (Fig. 4f, h).

Cytokine levels of IFN-γ, TNF-α, IL-2, and IL-6 were determined after co-culture of CFR T cells with SK-OV-3 or SK-BR-3 cells. When co-cultured with SK-OV-3 in the presence of herceptin, the levels of TNF-α and IL-6 significantly increased with 16s3, 32-8a and 64-8a CFR T cells; the increase of IFN-γ levels was observed with 16s3 and 64-8a CFR T cells. However, the IFN-γ released by the 32-8a CFR T cells and the IL-2 released by 16s3, 32-8a and 64-8a CFR T cells were not significantly increased (Fig. 4i).

When co-cultured with SK-BR-3 in the presence of herceptin, elevated levels of IFN-γ and TNF-α were observed with 16s3 and 64-8a CFR T cells; a significant increase in IL-2 levels was observed only with 64-8a CFR T cells; the levels of the above four cytokines were not significantly increased in the 32-8a CFR T cell group (Fig. 4j).

For these solid tumor cell lines, no significant cytotoxicity of the CFR T cells was observed when RTX or normal saline was added (Fig. 4a–e, i, j), proving again that the specificity of the CFR T cytotoxicity was directed by the specific MoAb.

### The CFR T cells confer cytotoxicity against the herceptin-resistant tumor cell line HCC1954

**Fig. 5:**
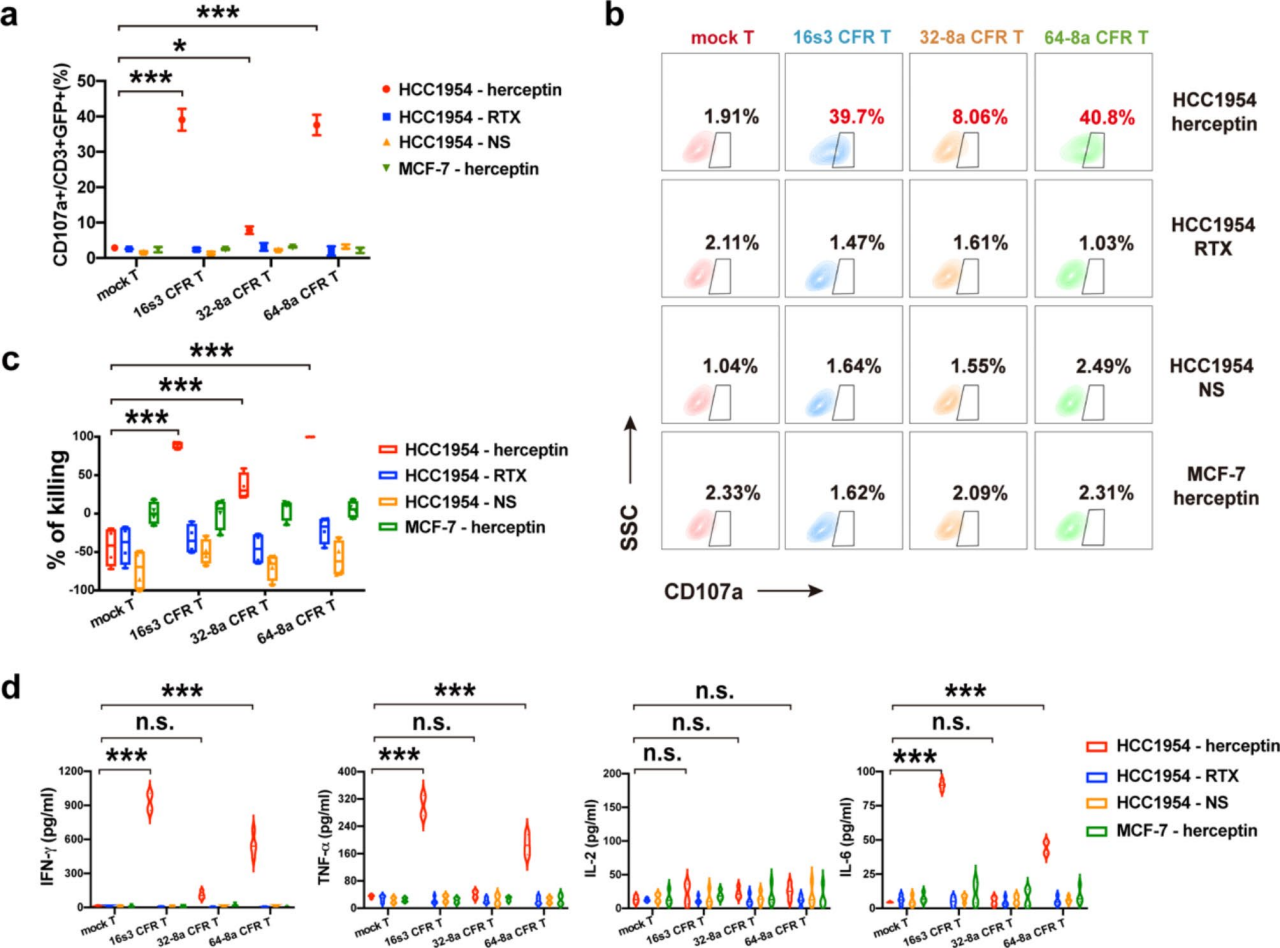
The cytotoxicity of CFR T cells against herceptin-resistant tumor cell line HCC1954. **a** The percentages of CD107a^+^ cells in 16s3, 32-8a, 64-8a CFR T and mock T cells co-cultured with HER2^+^ HCC1954 and MCF-7 (negative control target cells) in the presence of herceptin (1 µg/ml), rituximab (RTX) (1 µg/ml) or NS (*n* = 3; * * *, *P* < 0.001; *, *P* < 0.05). **b** The flow cytometry diagram showing the CD107a^+^ percentages of 16s3, 32-8a, 64-8a CFR T and mock T cells co-cultured with HCC1954 or MCF-7 in the presence of herceptin (1 µg/ml), RTX (1 µg/ml) or NS. **c** The HCC1954 and MCF-7 killing by 16s3, 32-8a, 64-8a CFR T and mock T cells in the presence of herceptin (1 µg/ml), RTX (1 µg/ml) or NS at 48 h (E: T = 2:1; *n* = 4; * * *, *P* < 0.001). **d** The IFN-γ, TNF-α, IL-2 and IL-6 levels secreted by 16s3, 32-8a, 64-8a CFR T and mock T cells in the presence of herceptin (1 µg/ml), RTX (1 µg/ml) or NS at 24 h (*n* = 4; * * *, *P* < 0.001; n.s., no significance)

Due to a PI3K mutation, HER2^+^ HCC1954 cell line exhibits inherent resistance to herceptin [47, 48].This resistance to herceptin was independently confirmed (Fig. S6b, c). Breast cancer cell line MCF-7 has no HER2 expression (Fig. S6a), and was used as a negative target cell control. After CFR T cells co-cultured with HCC1954 cells, the CD107a expression in the presence of herceptin increased significantly (Fig. 5a–b). The specific killing of HCC1954 by the CFR T cells in the presence of herceptin was also significantly increased in comparison to that by the mock T cells (Fig. 5c).

The cytokines (IFN-γ, TNF-α, and IL-6) secreted by both 16s3 and 64-8a CFR T cells in the presence of herceptin were significantly elevated (Fig. 5d). However, the IL-2 release by 16s3 and 64-8a CFR T cells in the presence of herceptin was not significantly increased. None of the four cytokines (IFN-γ, TNF-α, IL-2 and IL-6) were significantly increased with 32-8a CFR T cells in the presence of herceptin.

When RTX or normal saline was added to the co-culture, no significant cytotoxicity of the CFR T cells against the HER2^+^ HCC1954 was observed (Fig. 5a–d). When CFR T cells were co-cultured with the negative control MCF-7 cells in the presence of herceptin, no significant cytotoxicity was observed (Fig. 5a–d).

These results suggest that the 16s3 and 64-8a CFR T cells can confer specific cytotoxicity against HER2^+^ target cells which were resistant to herceptin alone.

### The impact of free human IgG on the cytotoxicity of the CFR T cells

To investigate the potential impact of free human IgG (hIgG) in the plasma on the CFR T cells, we evaluated the effect of hIgG on the cytotoxicity of the CFR T cells at both a physiological concentration (10 g/L) and a supraphysiological concentration (30 g/L).

**Fig. 6:**
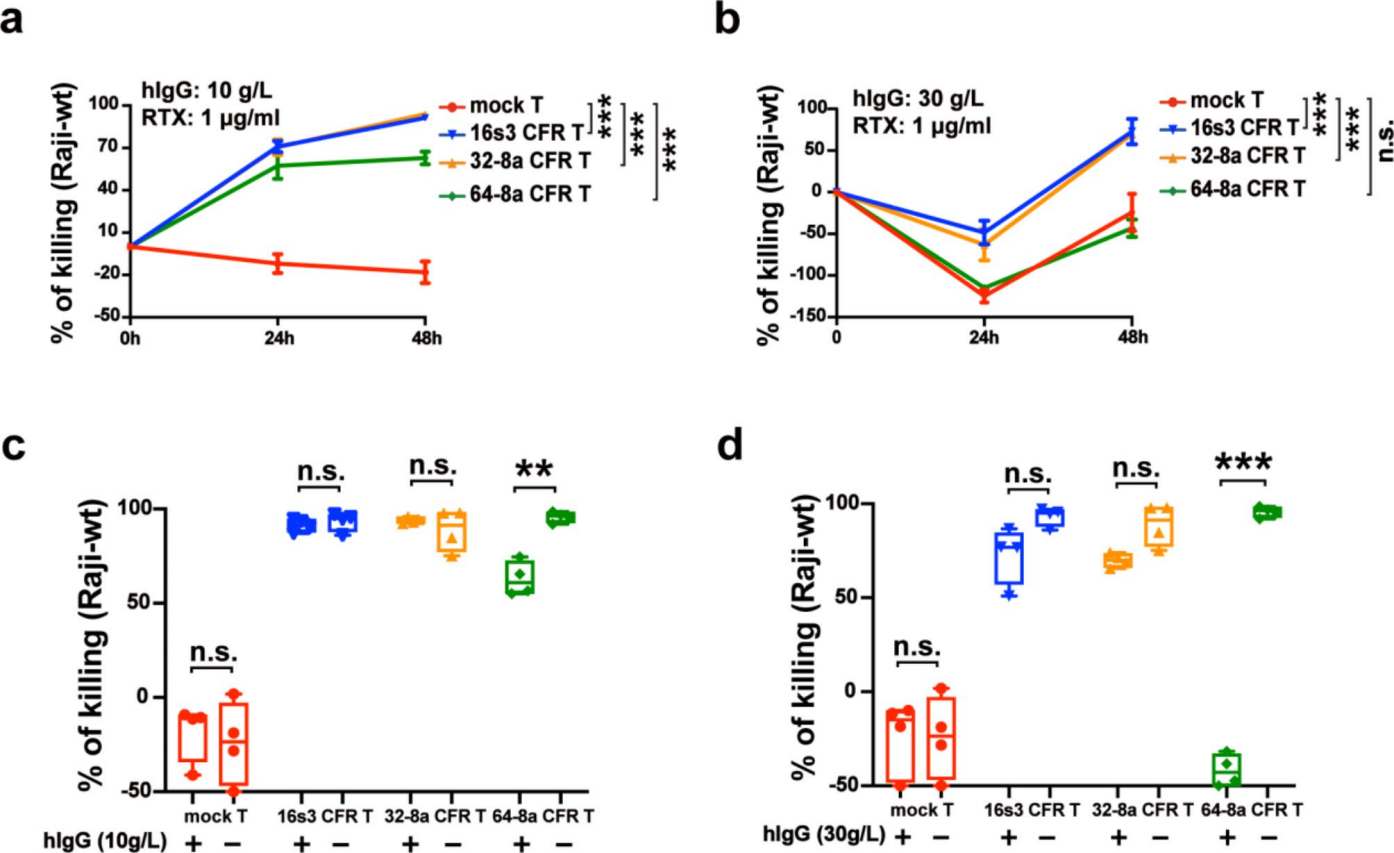
The impact of free hIgG on the CFR T cells. **a** The Raji-wt killing by 16s3, 32-8a, 64-8a CFR T and mock T cells in the presence of rituximab (RTX) (1 µg/ml) supplemented with hIgG at a physiological dose (10 g/L) (E: T = 2:1; *n* = 4; ***, *P* < 0.001). **b** The Raji-wt killing by 16s3, 32-8a, 64-8a CFR T and mock T cells in the presence of RTX (1 µg/ml) supplemented with hIgG at a supraphysiological dose (30 g/L) (E: T = 2:1; *n* = 4; ***, *P* < 0.001; n.s., no significance). **c** The comparison of Raji-wt killing by 16s3, 32-8a, 64-8a CFR T and mock T cells in the presence of RTX (1 µg/ml) supplemented with and without hIgG at a physiological dose (10 g/L) at 48 h (E: T = 2:1; *n* = 4; **, *P* < 0.01; n.s., no significance). **d** The comparison of Raji-wt killing by 16s3, 32-8a, 64-8a CFR T and mock T cells in the presence of RTX (1 µg/ml) supplemented with and without hIgG at a supraphysiological dose (30 g/L) at 48 h (E: T = 2:1; *n* = 4; ***, *P* < 0.001; n.s., no significance)

When co-cultured with Raji-wt, there was no significant effect on the cytotoxicity of 16s3 and 32-8a CFR T cells in the presence of 10 g/L hIgG (Fig. 6a, c). However, the cytotoxicity of 64-8a CFR T cells was partially but significantly inhibited compared to no hIgG addition (Fig. 6a, c). When exposed to 30 g/L hIgG, both 16s3 and 32-8a CFR T cells still exhibited significant tumor cell lysis (Fig. 6b, d). However, target cell lysis by 64-8a CFR T cells was completely inhibited (Fig. 6b, d). In addition, at physiological levels of hIgG (10 g/L), the 16s3, 32-8a and 64-8a CFR T cells still showed significant cytotoxic effects against HER2^+^ SK-OV-3 tumor cells (Fig.S7).

Overall, the physiological dose (10 g/L) and supraphysiological dose (30 g/L) of free hIgG have no significant impact on 16s3 and 32-8a CFR T cells but significant impact on the cytotoxicity of 64-8a CFR T cells.

### 16s3 CFR T cells exhibited superior long-term cytotoxicity to 32-8a CFR T cells in vitro

In the presence of herceptin, 16s3 CFR T cells exhibited significantly higher levels of CD107a, more target cell lysis and cytokine secretion than those of 32-8a CFR T cells (Fig. S8). No significant differences in CD20^+^ target cell lysis was observed between 16s3 CFR T cells and 32-8a CFR T cells in the presence of RTX (data not shown).

To further investigate the long-term cytotoxicity of 16s3 and 32-8a CFR T cells mediated by RTX, we implemented a repetitive antigen stimulation experiment (Fig. S9). To exclude the confounding effects of cytokine alterations, groups with or without cytokines were included. Half of the supernatant (500 µl) from the preceding round of co-culture was used to provide cytokines.

**Fig. 7:**
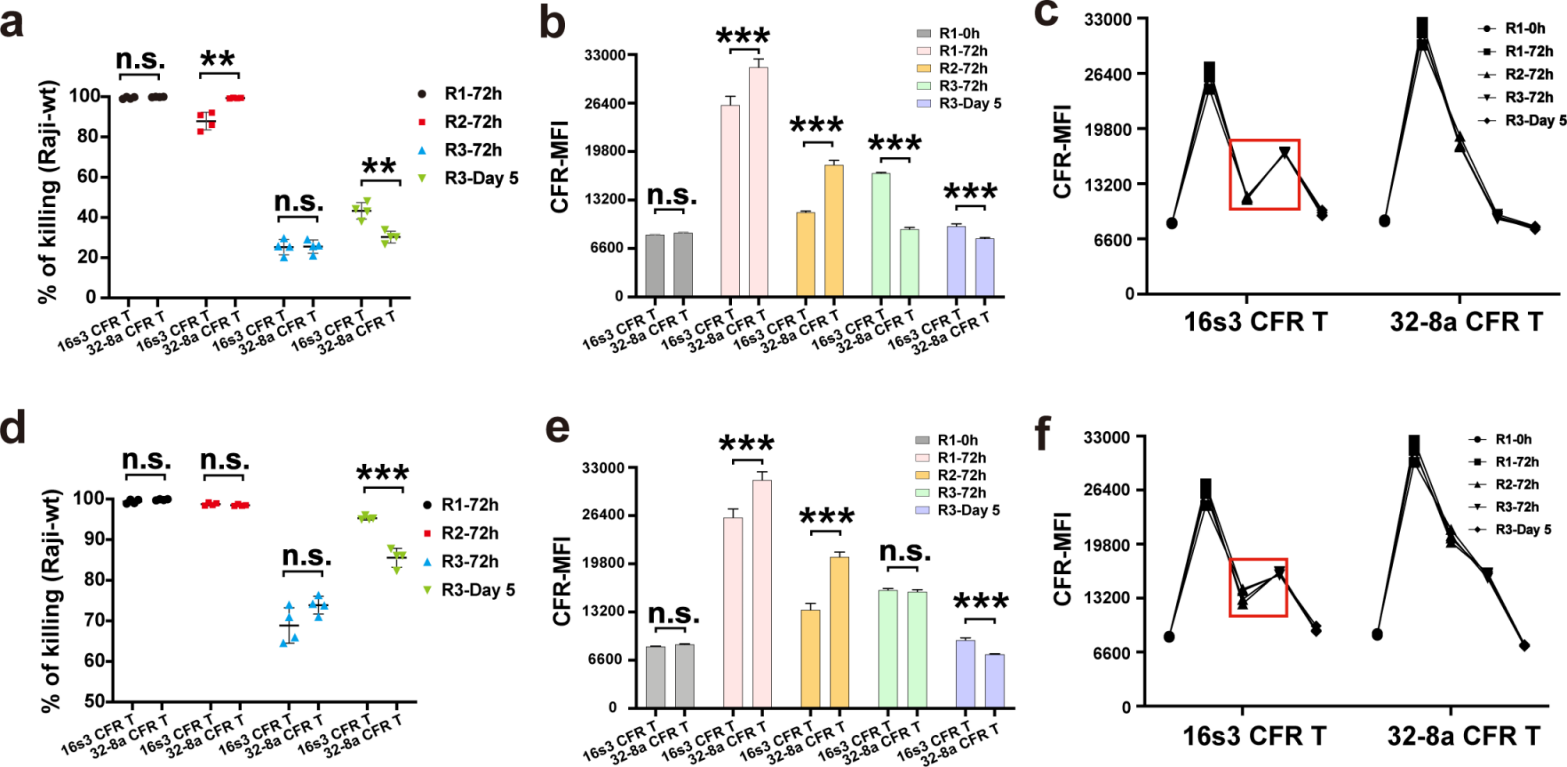
The comparison of rituximab (RTX)-mediated long-term cytotoxicity of 16s3 and 32-8a CFR T cells. **a** The Raji-wt killing by 16s3 and 32-8a CFR T cells at Round (R) 1–72 h, R2-72 h, R3-72 h, and R3-Day 5 of repetitive antigen stimulation without cytokines (*n* = 4; **, *P* < 0.01; n.s., no significance). **b** The comparison of the CFR-MFI of 16s3 and 32-8a CFR T cells at R1-0 h, R1-72 h, R2-72 h, R3-72 h, and R3-Day 5 without cytokines (*n* = 4; ***, *P* < 0.001; n.s., no significance). **c** The line chart showing the CFR-MFI of 16s3 and 32-8a CFR T cells at R1-0 h, R1-72 h, R2-72 h, R3-72 h, and R3-Day 5 without cytokines (*n* = 4). **d** The Raji-wt killing by 16s3 and 32-8a CFR T cells at R1-72 h, R2-72 h, R3-72 h, and R3-Day 5 with cytokines (*n* = 4; ***, *P* < 0.001; n.s., no significance). **e** The comparison of the CFR-MFI of 16s3 and 32-8a CFR T cells at R1-0 h, R1-72 h, R2-72 h, R3-72 h, and R3-Day 5 with cytokines (*n* = 4; ***, *P* < 0.001; n.s., no significance). **f** The line chart showing the CFR-MFI of 16s3 and 32-8a CFR T cells at R1-0 h, R1-72 h, R2-72 h, R3-72 h, and R3-Day 5 with cytokines (*n* = 4)

The long-term target lysis by 16s3 CFR T cells was significantly higher when compared to that of 32-8a CFR T cells at Round (R)3-Day 5, irrespective of the addition of cytokines (Fig. 7a, d). The peak value of the median fluorescence intensity (MFI) of 32-8a CFR T cells at R1-72 h was significantly higher than that of 16s3 CFR T cells in the groups without cytokines, potentially contributing to the enhanced target cell lysis observed at R2-72 h by 32-8a CFR T cells (Fig. 7a). However, the difference of target lysis between 16s3 and 32-8a CFR T cells was not found in the cytokine addition groups at R2-72 h (Fig. 7d). Notably, the CFR-MFI of 16s3 CFR T cells exhibited a significant increase from R2-72 h to R3-72 h, whereas no such trend was observed in 32-8a CFR T cells (Fig. 7c, f). Furthermore, the CFR-MFI of 16s3 CFR T cells was significantly higher than that of 32-8a CFR T cells at R3-Day 5 (Fig. 7b, e), which may partially account for the enhanced long-term cytotoxicity observed in the former.

Our findings suggest that 16s3 CFR T cells exhibited superior long-term anti-tumor potential in the presence of RTX in vitro. Therefore, 16s3 CFR T cells were selected to investigate the tumor clearance in mouse tumor transplantation models.

### Specific anti-tumor effect of 16s3 CFR T cells mediated by MoAbs in vivo

**Fig. 8:**
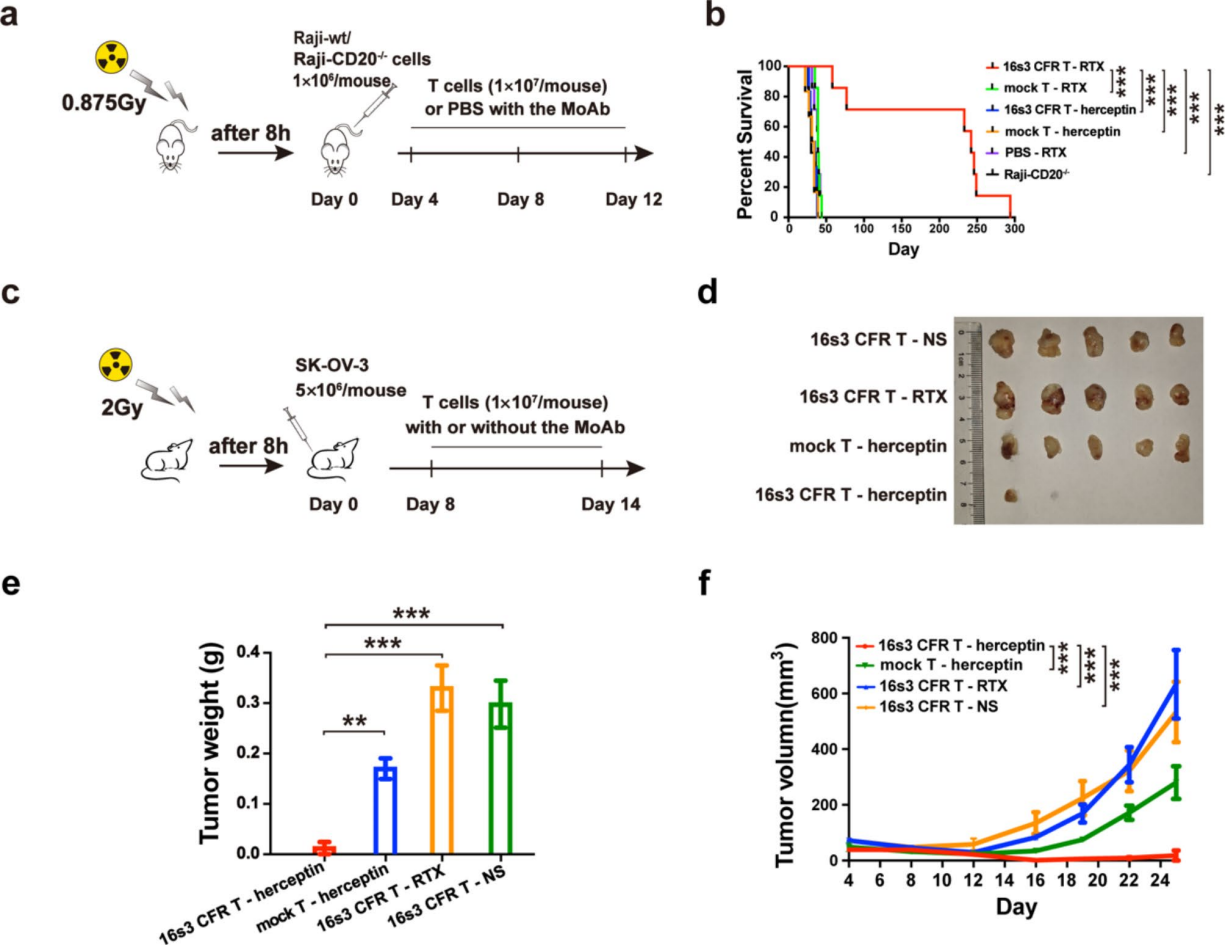
16s3 CFR T cells exhibit specific MoAb-mediated tumor clearance in mouse xenograft models. **a** The schematic diagram showing the transplantation and treatment of the human lymphoma model using Raji-wt or Raji-CD20^−/−^ cells. **b** The survival curves of mice in different treatment groups (***, *P* < 0.001). Mice transplanted with Raji-wt were treated respectively with 16s3 CFR T cells in the presence of rituximab (RTX) (16s3 CFR T - RTX) (*n* = 7), mock T - RTX (*n* = 7), 16s3 CFR T - herceptin (*n* = 6), mock T - herceptin (*n* = 6) or PBS - RTX (*n* = 7). Mice transplanted with Raji-CD20^−/−^ were treated with 16s3 CFR T - RTX (*n* = 7). **c** The schematic diagram of SK-OV-3 transplantation and treatment of the human ovarian model. **d** The tumor masses of the mice executed on Day 25 in different treatment groups (*n* = 5). **e** The tumor weight of mice in different treatment groups (*n* = 5; ***, *P* < 0.001; **, *P* < 0.01). **f** The tumor volumes of mice in different treatment groups from Day 4 to 25 (*n* = 5; ***, *P* < 0.001)

Two mouse models, the human lymphoma and human ovarian cancer xenograft models, were used for the evaluation of anti-tumor effect in vivo, and the corresponding schematic diagrams were shown in Fig. 8a, c.

For the mouse model transplanted with human lymphoma cells, the median survival of the 16s3 CFR T-RTX group (*n* = 7) was 242 days, which was significantly longer than those of the control groups [*P* < 0.001; mock T-RTX, 39 days (*n* = 7); PBS-RTX, 39 days (*n* = 7); Raji-CD20^−/−^, 30 days (*n* = 7); 16s3 CFR T-herceptin, 33 days (*n* = 6); and mock T-herceptin, 32.5 days (*n* = 6)] (Fig. 8b). Among the five control groups, the survival showed no significant difference (*P* > 0.05). For the mouse model transplanted with human ovarian cancer cells, euthanasia was performed on Day 25. The tumor masses were resected and examined (Fig. 8d). The average tumor weight of the 16s3 CFR T-herceptin group (*n* = 5) was 0.01 ± 0.03 g (95%CI: 0–0.05), which was significantly decreased when compared to those of the control groups [*P* < 0.01; mock T-herceptin, 0.17 ± 0.05 g (95%CI: 0.11–0.23) (*n* = 5); 16s3 CFR T-RTX, 0.33 ± 0.10 g (95%CI: 0.21–0.45) (*n* = 5); 16s3 CFR T-NS, 0.30 ± 0.10 g (95%CI: 0.17–0.43) (*n* = 5)] (Fig. 8e). Similarly, a significant reduction of tumor volume was observed in the 16s3 CFR T-herceptin group (Fig. 8f) (*P* < 0.001). It was noticed that the tumor weight and volume in the mock T-herceptin group was significantly decreased than those of 16s3 CFR T-RTX and 16s3 CFR T-NS groups (*P* < 0.05). Whereas no significant differences in tumor weight and volume were noted between the 16s3 CFR T-RTX and the 16s3 CFR T-NS groups (*P* > 0.05). 16s3 CFR T cells demonstrated a significant and specific MoAbs-mediated anti-tumor effect in the mouse xenograft models.

## Discussion

In this study, we engineered the CFR T cells expressing the extracellular region of three distinct forms of FcγR, CD16A, CD32A, and CD64, respectively. The anti-tumor effects of the CFR T cells are mediated by specific MoAbs such as RTX and herceptin. These specific anti-tumor activities were further confirmed in xenograft mouse models. These results suggested that the CFR T cells have the properties of universal CAR T cells, with the specificities bridged by different MoAbs. With many MoAbs in clinical uses, this makes the CFR T cells applicable for treatment of various cancers.

There is a concern that high concentrations of endogenous free IgG may interfere or quench the cytotoxicities of the CFR T cells in vivo. We showed that only the cytotoxicity of 64-8a CFR T cells was significantly attenuated by high concentrations of free hIgG in vitro. 16s3 CFR T cells were chosen for further assay in mouse models and exhibited significant anti-tumor activity.

A previous study showed the binding of plasma IgG to CFR T cells. The study demonstrated that there was no difference in MoAb binding to 16CAR and 32CAR, though a moderate reduction in RTX binding to 64CAR T cells was observed in human serum [36]. Even though the effects of free hIgG on the cytotoxicity of CFR T cells were not discussed in this study, this and our study nevertheless reached similar conclusion that free polyclonal IgG in serum had negligible effect on targeted cytotoxicity of CD16 and CD32 engineered CFR T cells.

A study by Rasoulouniriana et al. proposed that 64 CFR T cells exhibit superior persistence and anti-tumor effects compared to 16 and 32 CFR T cells [49]. However, the impact of free hIgG on the cytotoxicity of 64 CFR T cells was not discussed in their study. Our findings, along with those of Zhu et al., suggest that free hIgG inhibits the cytotoxicity of 64 CFR T cells [36]. Variations between different individuals and disease treatment status may lead to dramatic fluctuations in plasma IgG concentrations, which could result in uncertainty regarding the anti-tumor efficacy of 64 CFR T cells.

Our results suggest that the cytotoxicity of herceptin-mediated 16s3 CFR T cells was significantly superior to that of 32-8a CFR T cells. This observation is consistent with the findings reported by Arriga et al., who demonstrated a significant clearance of colorectal cancer cells by CD16^158V^ chimeric receptor T cells mediated by cetuximab, while no such effect was observed with CD32^131H^ chimeric receptor T cells [50]. Herceptin is a humanized IgG1 monoclonal antibody, and according to existing evidence, its affinity for FcγRIIA-131H is not lower than that for FcγRIIIA-158V [36, 51]. Meanwhile, we adjusted the viral titer to ensure that the expression levels of the three CFRs on T cells were comparable. Therefore, the difference in cytotoxicity induced by herceptin between 16s3 and 32-8a CFR T cells may not be due to differences in affinity or expression. We speculate that this difference arises from the “differential matching” between cell function and the function of the Fcγ receptors. The properties of different FcγRs have been extensively reviewed elsewhere [16, 52–54]. In brief, CD16A activation can trigger the ADCC of NK cells and the ADCC and ADCP of macrophages. Since activated NK cells share very similar biological functions to activated CTLs, such as cytotoxic granule secretion and cytokine release [55], 16s3 CFR can maximize the cytotoxic function of T cells. However, CD32A activation can initiate the ADCC and ADCP of macrophages. Since activated CTL cells cannot undergo phagocytosis like macrophages, in other words, CD32A-mediated functions do not fully match T cell functionality. Therefore, 32-8a CFR overexpressed on T cells may result in impaired function due to the inability to initiate ADCP. Although not to the maximum extent, 32-8a CFR T cells still exhibit incomplete cytotoxicity or may require a longer priming to fully activate T cells since they can still trigger ADCC partially. In cases of insufficient T cell activation, such as when targeting solid tumor cells, this functional compromise may be further exacerbated.

The cytotoxicity of CAR T cells exposed to long-term and repetitive antigens has been reported to be significantly compromised, yet prolonged persistence of CAR T cells are well known to be required for enhanced and durable in vivo activity [56–58]. Cui et al. also confirmed that long-term maintenance of CFR T cell effector function is associated with improved in vivo tumor clearance [37]. Consistent with the findings, our findings indicate that 16s3 CFR T cells exhibit enhanced long-term cytotoxicity upon repetitive antigenic stimulation.

16s3 CFR T cells were minimally disturbed by plasma IgG compared to 64-8a CFR T cells and exhibited long-lasting and superior anti-tumor effects mediated by RTX and herceptin compared with 32-8a CFR T cells. In lymphoma and ovarian cancer mouse xenograft models, 16s3 CFR T cells showed significant tumor clearance in vivo, and no significant non-specific effects were found. The 16s3 CFR T cell has demonstrated its potential as a universal platform, making it suitable for further development in clinical trials.

## Electronic supplementary material

Below is the link to the electronic supplementary material.

Supplementary Material 1

Supplementary Material 2

Supplementary Material 3

Supplementary Material 4

Supplementary Material 5

Supplementary Material 6

Supplementary Material 7

Supplementary Material 8

Supplementary Material 9

## Data Availability

No datasets were generated or analysed during the current study.
