# Supplementary material for: Construction and characterization of chimeric FcγR T cells for universal T cell therapy
Source: Exp Hematol Oncol. 2025 Jan 15;14:6. doi: 10.1186/s40164-025-00595-x (PMC11734343; doi:10.1186/s40164-025-00595-x)
Supplement: Supplementary file 1 — Supplementary Material 1 [file 40164_2025_595_MOESM1_ESM.docx]

**Fig. S1**

**
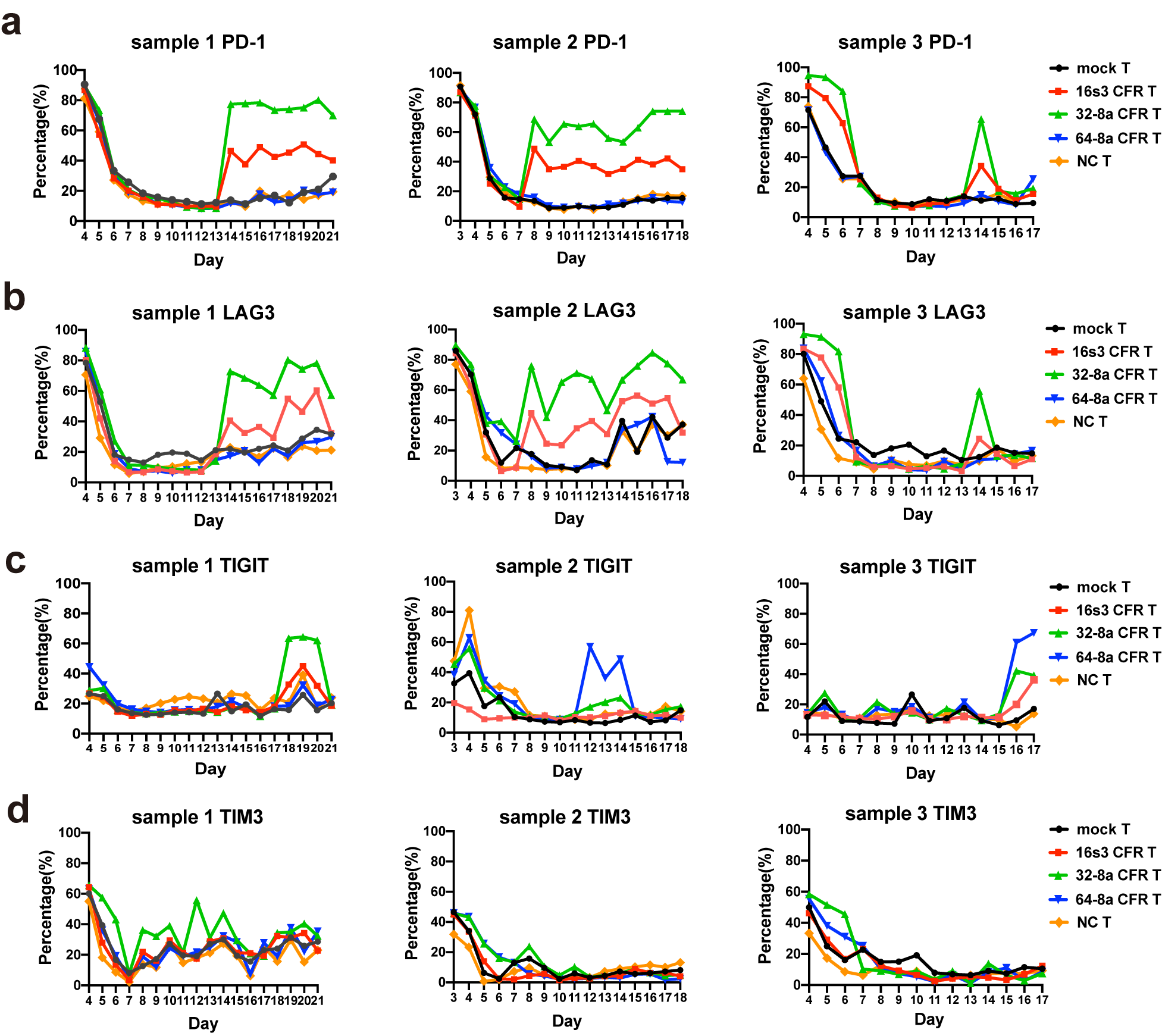
**

**Supplementary Figure 1.** **The expression of exhaustion markers on T cells.** The expression levels of PD-1 (**a**), LAG3 (**b**), TIGIT (**c**), and TIM3 (**d**) on 16s3, 32-8a, 64-8a CFR T, mock T and un-transduced negative control (NC) T cells derived from three different samples during culture.
