# Supplementary material for: Construction and characterization of chimeric FcγR T cells for universal T cell therapy
Source: Exp Hematol Oncol. 2025 Jan 15;14:6. doi: 10.1186/s40164-025-00595-x (PMC11734343; doi:10.1186/s40164-025-00595-x)
Supplement: Supplementary file 2 — Supplementary Material 2 [file 40164_2025_595_MOESM2_ESM.docx]

**Fig. S2**


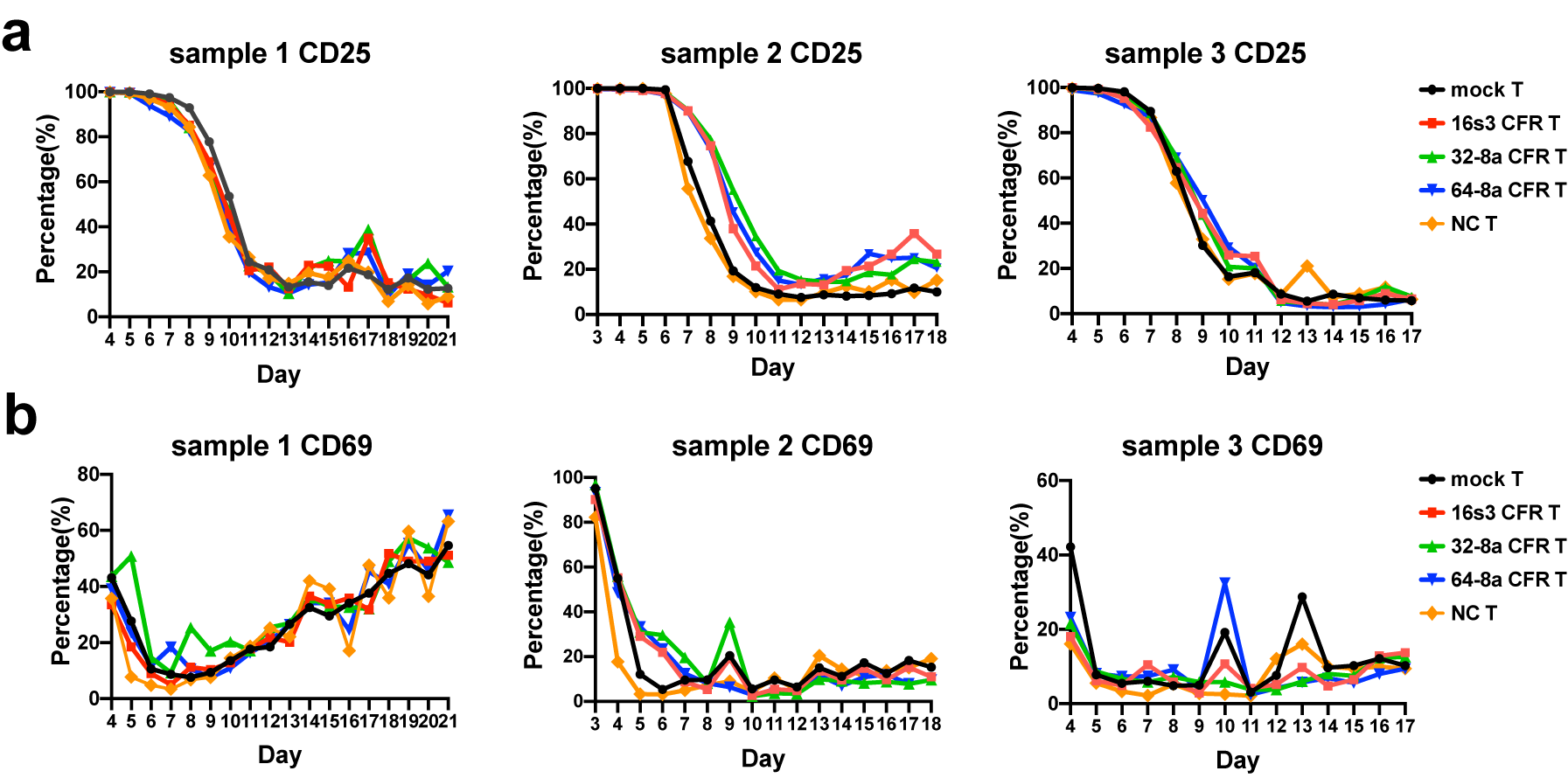


**Supplementary Figure 2. The expression of activation markers on T cells.** The expression levels of CD25 (**a**) and CD69 (**b**) on 16s3, 32-8a, 64-8a CFR T, mock T and NC T cells derived from three different samples during culture.
