# Supplementary material for: Construction and characterization of chimeric FcγR T cells for universal T cell therapy
Source: Exp Hematol Oncol. 2025 Jan 15;14:6. doi: 10.1186/s40164-025-00595-x (PMC11734343; doi:10.1186/s40164-025-00595-x)
Supplement: Supplementary file 3 — Supplementary Material 3 [file 40164_2025_595_MOESM3_ESM.docx]

**Fig. S4**


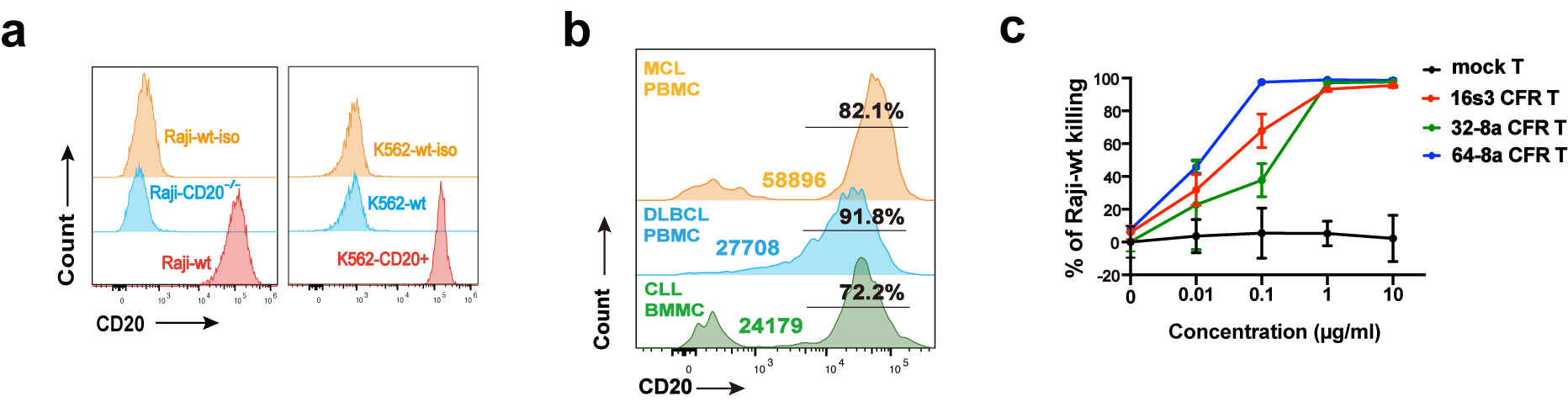


**Supplementary Figure 4.** **CD20 antigen expression, specific killing mediated by different concentrations of RTX. a** The CD20 expression of Raji-wt, Raji-CD20^-/-^, K562-wt and K562-CD20^+^ cells. **b** The percentages and MFI of CD20 expression in primary cells collected from patients. **c** The Raji-wt killing by 16s3, 32-8a, 64-8a CFR T and mock T cells in the presence of RTX at different concentrations (0, 0.01, 0.1, 1, and 10 μg/ml) at 24 hours (n = 3).
