# Supplementary material for: Construction and characterization of chimeric FcγR T cells for universal T cell therapy
Source: Exp Hematol Oncol. 2025 Jan 15;14:6. doi: 10.1186/s40164-025-00595-x (PMC11734343; doi:10.1186/s40164-025-00595-x)
Supplement: Supplementary file 4 — Supplementary Material 4 [file 40164_2025_595_MOESM4_ESM.docx]

**Fig. S5**


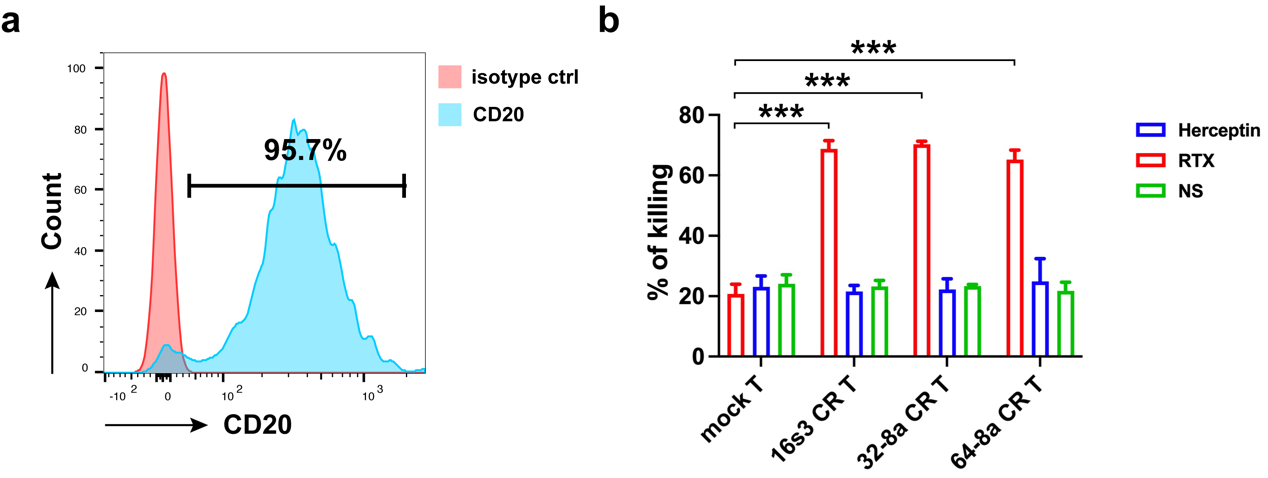


**Supplementary Figure 5.** **The rituximab (RTX)-mediated elimination of CD20^+^ normal B cell by the CFR T cells. a** The CD20 expression of normal B cells isolated from peripheral blood. **b** The killing percentages of normal CD20^+^ B cells by 16s3, 32-8a, 64-8a CFR T and mock T cells in the presence of RTX (1 μg/ml), herceptin (1 μg/ml) or normal saline (NS) at 24h (E:T = 2:1; n = 3; ***, *P* < 0.001).
