# Supplementary material for: Construction and characterization of chimeric FcγR T cells for universal T cell therapy
Source: Exp Hematol Oncol. 2025 Jan 15;14:6. doi: 10.1186/s40164-025-00595-x (PMC11734343; doi:10.1186/s40164-025-00595-x)
Supplement: Supplementary file 5 — Supplementary Material 5 [file 40164_2025_595_MOESM5_ESM.docx]

**Fig. S7**


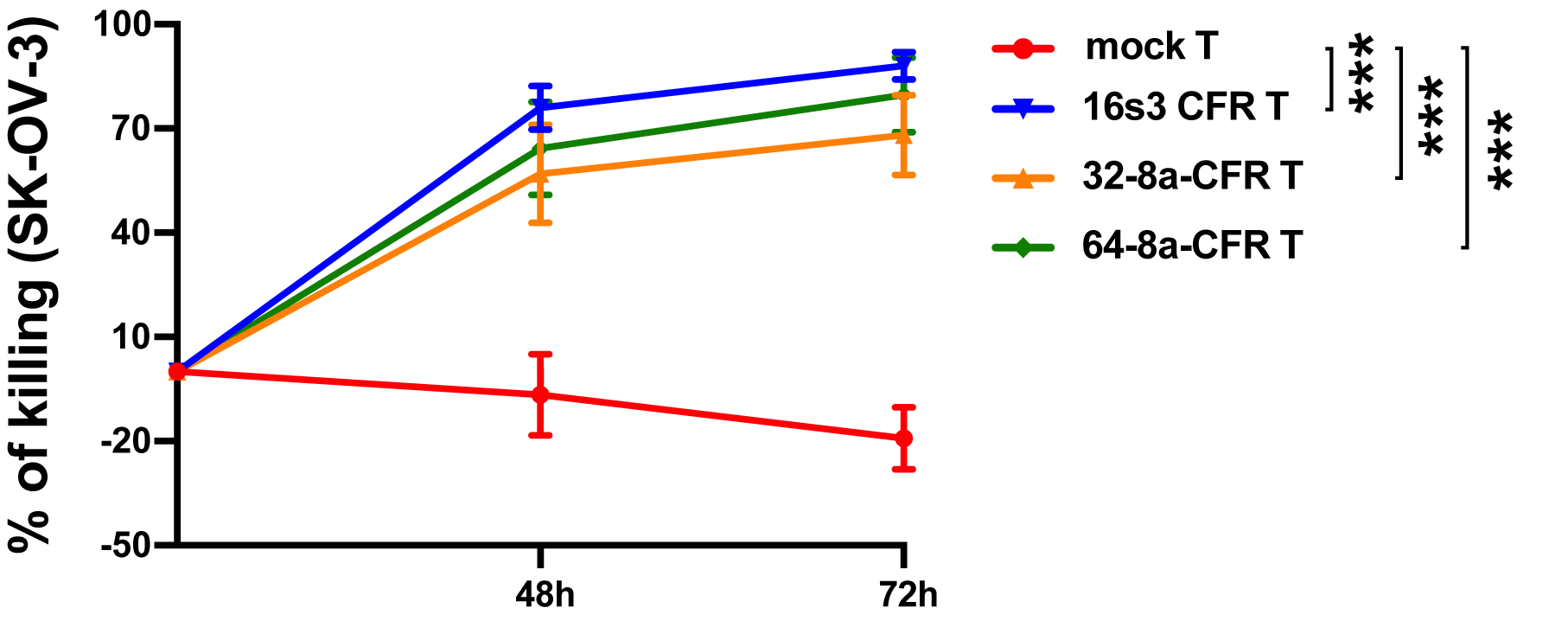


**Supplementary Figure 7.** **The specific cytotoxicity of CFR T cells towards SK-OV-3 mediated by herceptin under a physiological concentration hIgG.** The SK-OV-3 killing by 16s3, 32-8a, 64-8a CFR T and mock T cells in the presence of herceptin (1 μg/ml) supplemented with hIgG at a physiological dose (10 g/L) at 72 hours (E:T = 2:1; n = 3; ***, *P* < 0.001).
