# Supplementary material for: Construction and characterization of chimeric FcγR T cells for universal T cell therapy
Source: Exp Hematol Oncol. 2025 Jan 15;14:6. doi: 10.1186/s40164-025-00595-x (PMC11734343; doi:10.1186/s40164-025-00595-x)
Supplement: Supplementary file 6 — Supplementary Material 6 [file 40164_2025_595_MOESM6_ESM.docx]

**Fig. S6**


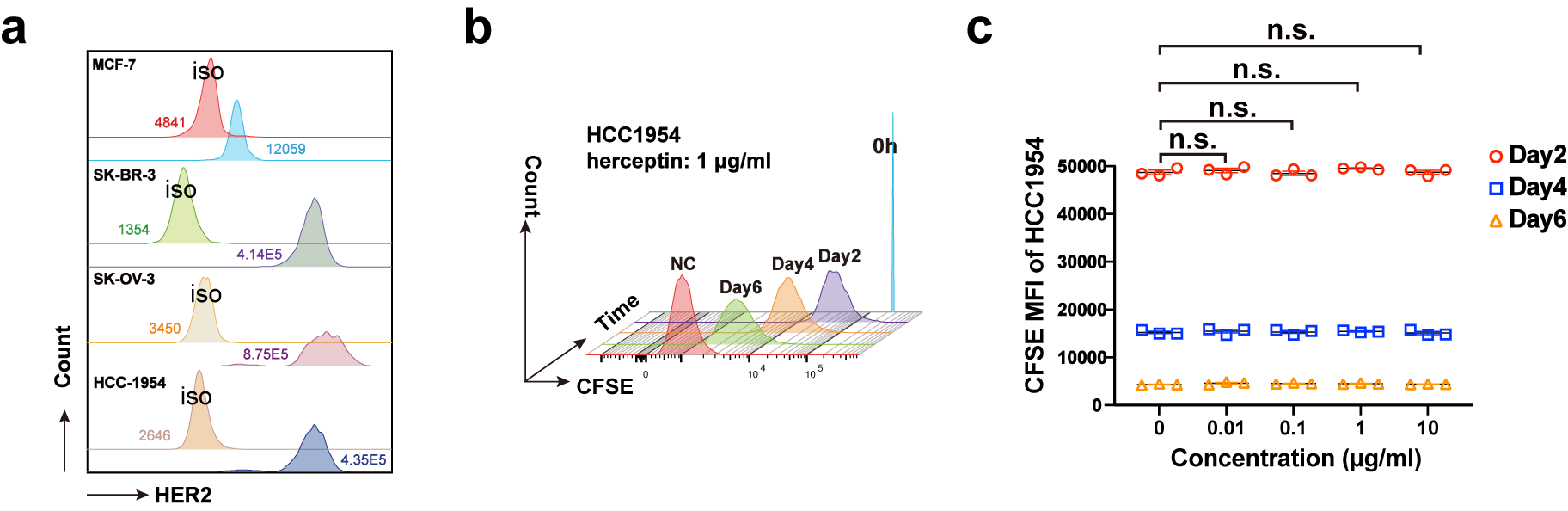


**Supplementary Figure 6.** **HER2 antigen expression and the impact of herceptin on HCC1954** **proliferation. a** The the MFI of HER2 antigen on MCF-7, SK-BR-3, SK-OV-3 and HCC1954 cell lines. **b** The histogram by flow cytometry illustrating the CFSE attenuation of HCC1954 cells in the presence of herceptin (1 μg/ml) on Day 2, 4, 6 following CFSE staining (Day 0). **c** The CFSE MFI attenuation of HCC1954 cells during culture in the presence of different concentrations of herceptin (0, 0.01, 0.1, 1, and 10 μg/ml) on Day 2, 4, and 6 (n = 3; n.s., no significance).
