# Supplementary material for: Construction and characterization of chimeric FcγR T cells for universal T cell therapy
Source: Exp Hematol Oncol. 2025 Jan 15;14:6. doi: 10.1186/s40164-025-00595-x (PMC11734343; doi:10.1186/s40164-025-00595-x)
Supplement: Supplementary file 7 — Supplementary Material 7 [file 40164_2025_595_MOESM7_ESM.docx]

**Fig. S3**


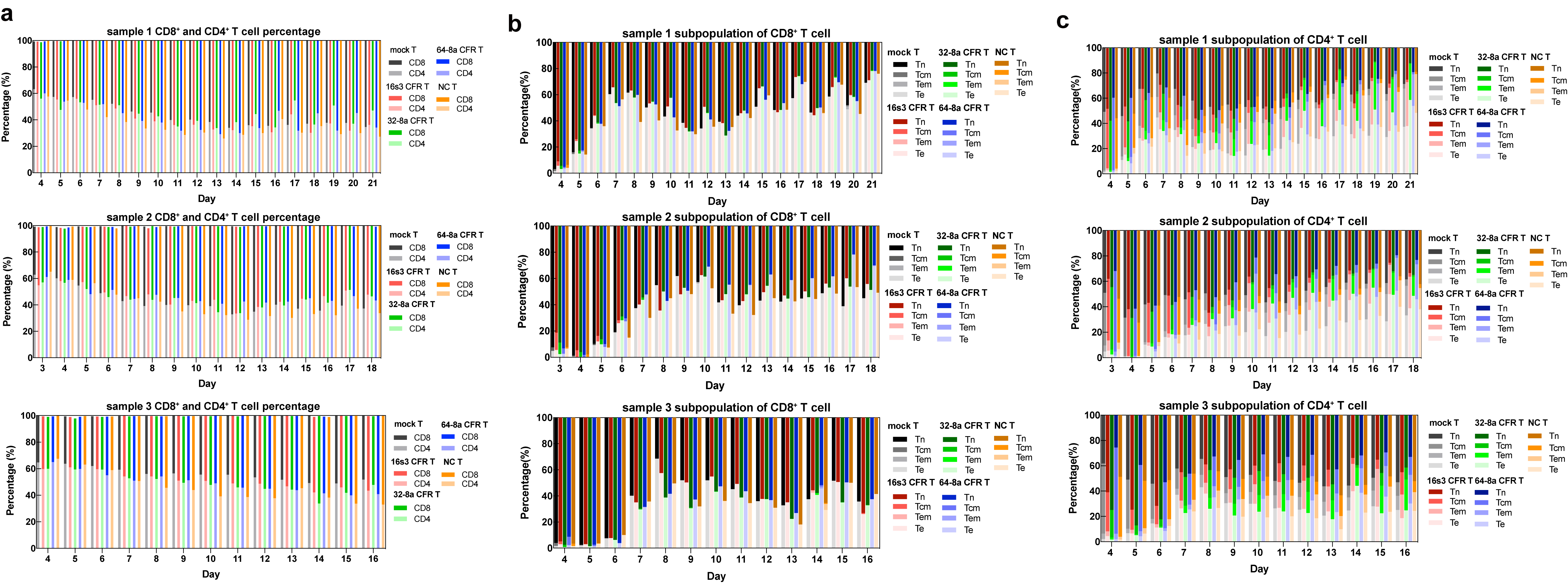


**Supplementary Figure 3.** **The subpopulation differentiation of T cells after transduction.** **a** The percentages of CD8^+^ and CD4^+^ subpopulations in 16s3, 32-8a, 64-8a CFR T, mock T and NC T cells during culture. Different subpopulations of T cells were distinguished by distinct colors; dark color represented CD8^+^ T cells while light color indicated CD4^+^ T cells. **b, c** The subpopulation percentages of CD8^+^ (**b**), CD4^+^ (**c**) naive T cells (Tn), central memory T cells (Tcm), effector memory T cells (Tem) and terminal effector T cells (Te) in 16s3, 32-8a, 64-8a CFR T, mock T and NC T cells during culture. Different subpopulations of T cells were represented by distinct colors. The transition from dark to light color indicated the differentiation from Tn, Tcm, Tem to Te as indicated.
