# Supplementary material for: Construction and characterization of chimeric FcγR T cells for universal T cell therapy
Source: Exp Hematol Oncol. 2025 Jan 15;14:6. doi: 10.1186/s40164-025-00595-x (PMC11734343; doi:10.1186/s40164-025-00595-x)
Supplement: Supplementary file 8 — Supplementary Material 8 [file 40164_2025_595_MOESM8_ESM.docx]

**Fig. S9**


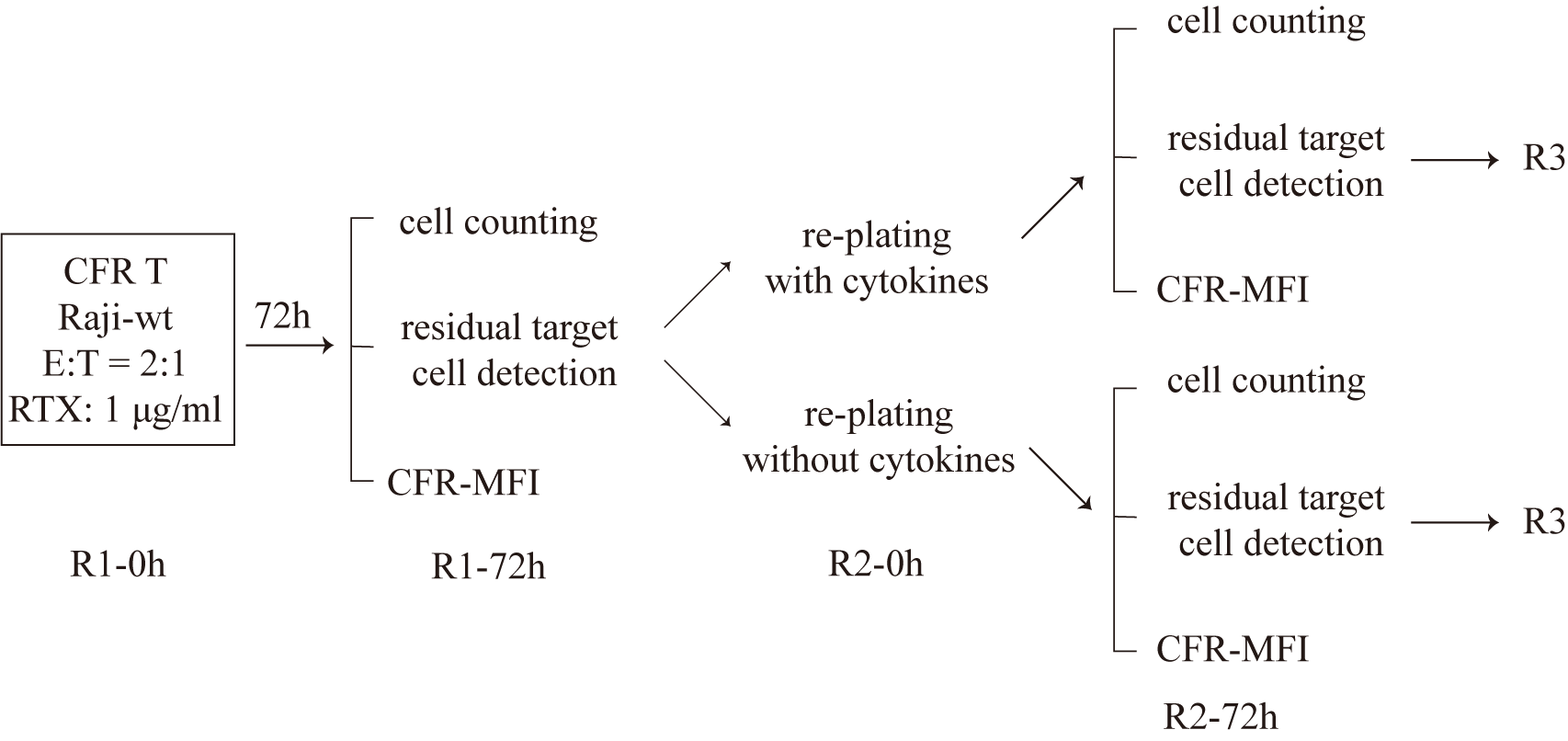


**Supplementary Figure 9.** **The schematic flow of the repetitive antigen stimulation of 16s3 and 32-8a CFR T cells by Raji-wt cells.**
