# Supplementary material for: Construction and characterization of chimeric FcγR T cells for universal T cell therapy
Source: Exp Hematol Oncol. 2025 Jan 15;14:6. doi: 10.1186/s40164-025-00595-x (PMC11734343; doi:10.1186/s40164-025-00595-x)
Supplement: Supplementary file 9 — Supplementary Material 9 [file 40164_2025_595_MOESM9_ESM.docx]

**Fig. S8**


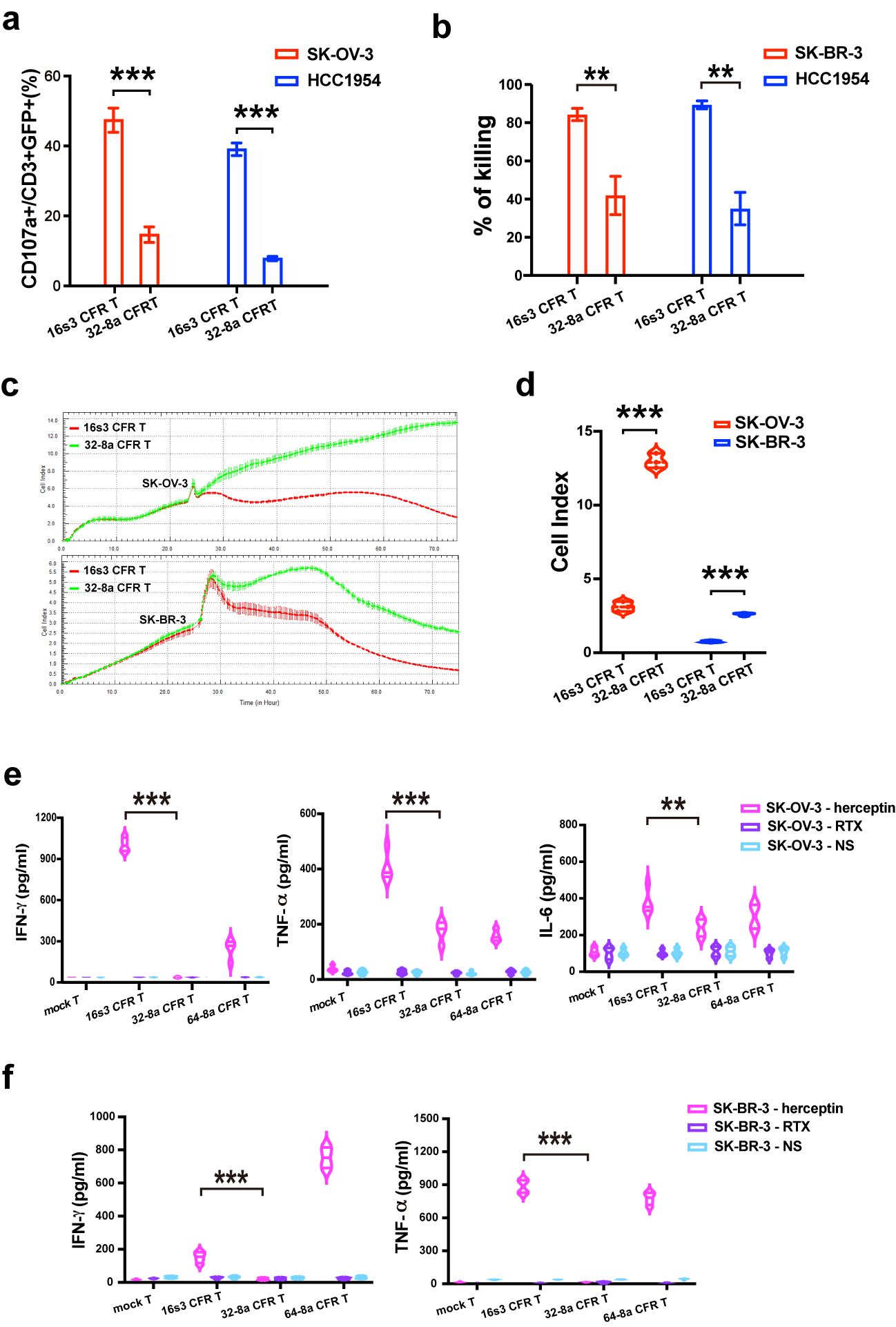


**Supplementary Figure 8.** **The comparison of herceptin-mediated cytotoxicity of 16s3 and 32-8a CFR T cells. a** The comparison of the percentages of CD107a^+^ cells in 16s3 and 32-8a CFR T cells co-cultured with SK-OV-3 (n = 5) and HCC1954 (n = 3) in the presence of herceptin (1 μg/ml) (* * *, *P* < 0.001). **b** The comparison of SK-BR-3 (n = 5) and HCC1954 (n = 3) lysis by 16s3 and 32-8a CFR T cells in the presence of herceptin (1 μg/ml) at 48 hours (E:T = 2:1; * *, *P* < 0.01). **c** The CI values displayed by the RTCA system of SK-OV-3 and SK-BR-3 co-cultured with 16s3 and 32-8a CFR T cells at the E:T of 2:1 in the presence of herceptin (1 μg/ml). **d** The comparison of CI values of SK-OV-3 (n = 3) and SK-BR-3 (n = 4) co-cultured with 16s3 and 32-8a CFR T cells (E:T = 2:1; 48 hours; * * *, *P* < 0.001). **e** The comparison of IFN-γ, TNF-α, and IL-6 levels secreted by 16s3 and 32-8a CFR T cells co-cultured with SK-OV-3 at 24 hours (n = 4; * * *, *P* < 0.001; * *, *P* < 0.01). **f** The comparison of IFN-γ and TNF-α levels secreted by 16s3 and 32-8a CFR T cells co-cultured with SK-BR-3 at 24 hours (n = 4; * * *, *P* < 0.001).
